# Pathogenesis and treatment of multiple myeloma

**DOI:** 10.1002/mco2.146

**Published:** 2022-06-02

**Authors:** Peipei Yang, Ying Qu, Mengyao Wang, Bingyang Chu, Wen Chen, Yuhuan Zheng, Ting Niu, Zhiyong Qian

**Affiliations:** ^1^ Department of Hematology and Institute of Hematology, State Key Laboratory of Biotherapy and Cancer Center West China Hospital Sichuan University Chengdu Sichuan China

**Keywords:** multiple myeloma, nanomedicine, pathogenesis, treatment

## Abstract

Multiple myeloma (MM) is the second‐ranking malignancy in hematological tumors. The pathogenesis of MM is complex with high heterogeneity, and the development of the disease is a multistep process. Chromosomal translocations, aneuploidy, genetic mutations, and epigenetic aberrations are essential in disease initiation and progression. The correlation between MM cells and the bone marrow microenvironment is associated with the survival, progression, migration, and drug resistance of MM cells. In recent decades, there has been a significant change in the paradigm for the management of MM. With the development of proteasome inhibitors, immunomodulatory drugs, monoclonal antibodies, chimeric antigen receptor T‐cell therapies, and novel agents, the survival of MM patients has been significantly improved. In addition, nanotechnology acts as both a nanocarrier and a treatment tool for MM. The properties and responsive conditions of nanomedicine can be tailored to reach different goals. Nanomedicine with a precise targeting property has offered great potential for drug delivery and assisted in tumor immunotherapy. In this review, we summarize the pathogenesis and current treatment options of MM, then overview recent advances in nanomedicine‐based systems, aiming to provide more insights into the treatment of MM.

## Graphical abstract

Multiple myeloma (MM) is the second‐ranking malignancy in hematological tumors. There has been a significant change in the paradigm for the management of MM. In this review, we summarize the pathogenesis and current treatment options of MM, then overview recent advances in nanomedicine‐based systems, aiming to provide more insights into the treatment of MM.

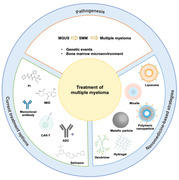

## 1 INTRODUCTION

**FIGURE 1:**
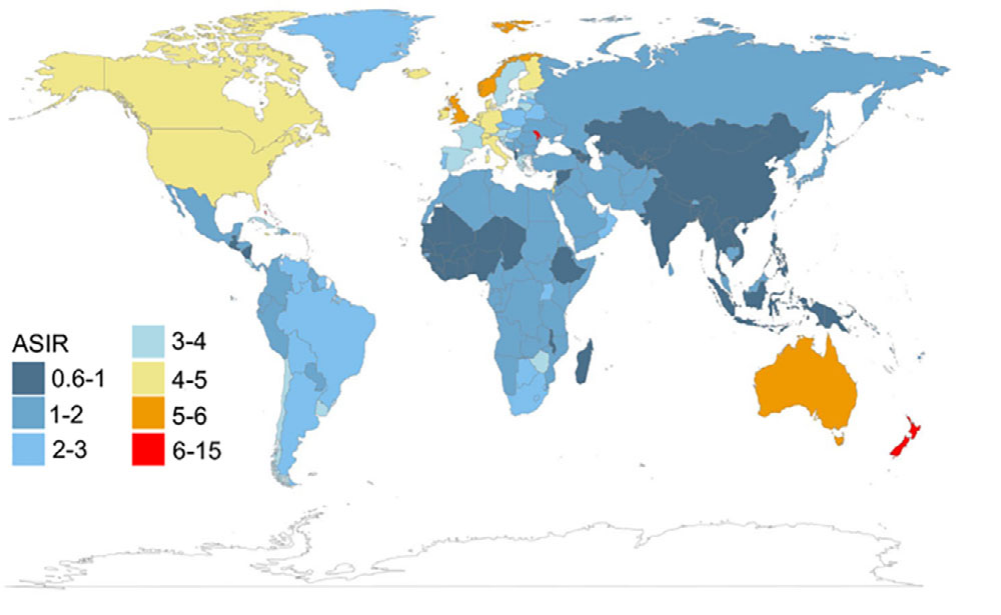
Age‐standardized rates of incidence of multiple myeloma worldwide in 2019. ASIR, age‐standardized incidence rate (Copyright 2021, Springer Nature^3^)

As the second‐ranking hematological malignancy, multiple myeloma (MM) is a disease characterized by clonal expansion of malignant plasma cells that accumulate in the bone marrow (BM).^1^ The latest cancer statistics indicated that in 2021, there were 34,920 estimated new cases of MM in the United States, with an estimated 12,410 deaths.^2^ The disease is prevalent in people over 65 years old, and more than half of the patients are men. In 2019, there were 155,688 MM patients worldwide, with an increased global burden (Figure 1). Besides, MM had a higher age‐standardized incidence rate and age‐standardized death rate in more developed countries.^3^

The etiology of MM is not clear, while environmental exposures and genetic events may be risk factors. In almost all patients, MM evolves from an asymptomatic premalignant stage termed monoclonal gammopathy of undetermined significance (MGUS). The definition of MGUS is less than 30 g/L of M protein, less than 10% of clonal BM plasma cells, and the absence of symptoms related to MM.^4^ Another asymptomatic but more advanced stage refers to smoldering MM (SMM). The median time to progression from SMM to active MM is around 5 years.^5^ The risk of progression relates to the proportion of plasma cells in the BM and the serum monoclonal protein level at diagnosis. The genetic events increase the progression rate from MGUS to SMM and then to MM.^6^, ^7^

The proliferation of monoclonal plasma cells in the BM impedes the normal course of blood cells and leads to anemia. In addition, malignant plasma cells secrete monoclonal immunoglobulin, the so‐called paraprotein or M‐protein, and infiltrate other vital organs. Bone pain is one of the symbolized manifestations of MM, including spine, chest, and long bones, due to increased activity of osteoclasts and enhanced bone resorption.^8^ The lytic lesions and fractures of bones lead to high blood calcium levels. The MM defining events, hypercalcemia, renal insufficiency, anemia, and bone lesions, are commonly referred to as the “CRAB” feature.^9^ Besides, myeloma is always associated with hyperviscosity, amyloidosis, fatigue, and recurrent infections.^10^ And the presence of extramedullary foci indicates a more aggressive situation.

**FIGURE 2:**
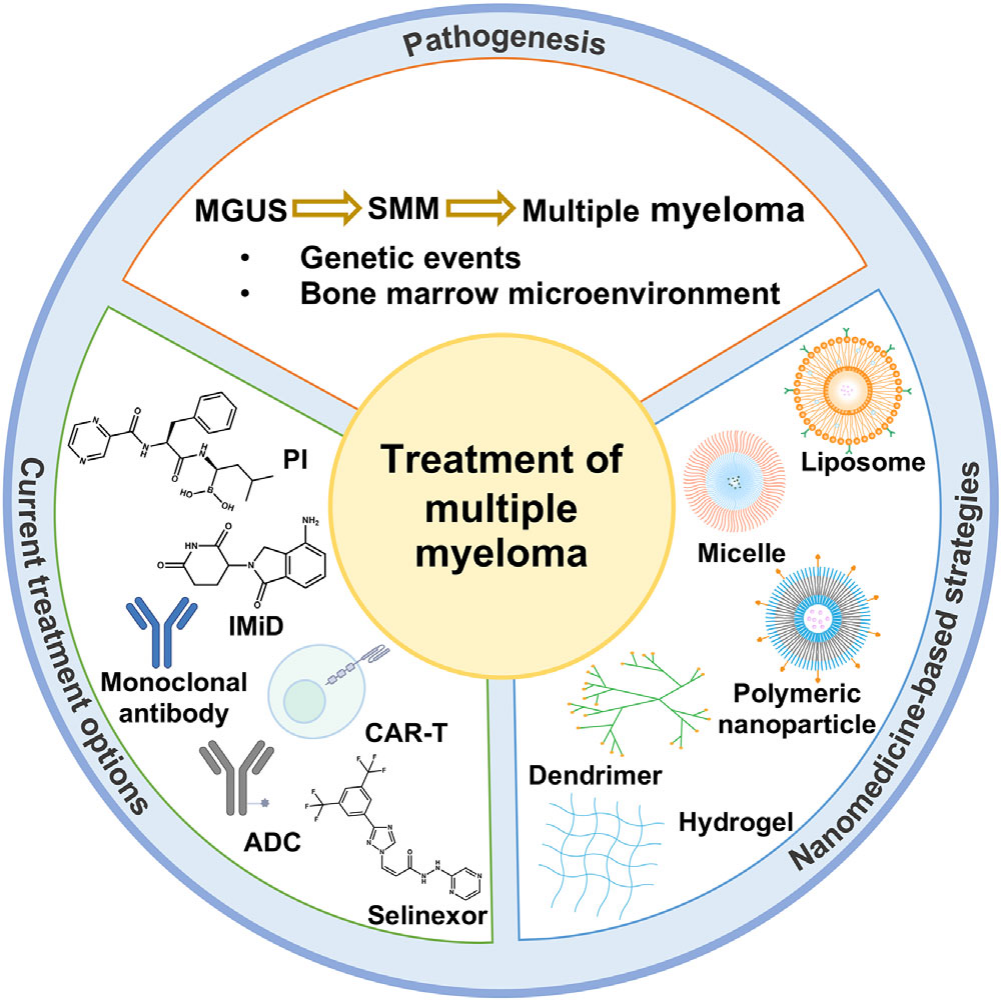
Schematic illustration of treatment options and nanomedicine‐based strategies for multiple myeloma. ADC, antibody‐drug conjugate; CAR‐T, chimeric antigen receptor T cell; IMiD, immunomodulatory drug; MGUS, monoclonal gammopathy of undetermined significance; PI, proteasome inhibitor; SMM, smoldering multiple myeloma;

Over the last decades, there has been increased attention on disease evolution and pathogenesis. Significant changes have also been made in the paradigm for the management of MM. A good variety of agents can be offered to MM patients at different phases. In this review, we summarize the pathogenesis and current treatment options of MM, then overview recent advances in nanomedicine‐based systems, aiming to provide more insights into the treatment of MM (Figure 2).

## 2 PATHOGENESIS OF MULTIPLE MYELOMA

### 2.1 Genetic alterations

The pathogenesis of MM is complex with high heterogeneity, and the development of the disease is a multistep process. Chromosomal translocations, aneuploidy, genetic mutations, and epigenetic aberrations are essential in disease initiation and progression.^11^ The initiating events are thought to occur in the germinal center during the procedure of class switching and somatic hypermutation.^12^ Double‐strand DNA breaks and fuses with other breaks in the genome, leading to aberrant fusions and chromosomal translocations. And translocations involving oncogenes can lead to pathological states including MGUS, SMM, and MM. The identified translocations involve the immunoglobulin heavy chain (IgH) gene loci and a set of recurrent partner genes. A vast majority of chromosomal translocations are related to the IgH chain locus at chromosome 14, resulting in specific oncogenes falling under the control of the IgH enhancer. The t(11;14) translocation results in a high prevalence of CCND1, which encodes cyclin D1 and is essential for cell cycle progress.^13^ The t(4;14) leads to increased expression of NSD2 and FGFR3, and is identified in 10 to 15% of MM patients.^14^ The overall survival (OS) of patients with t(4;14) was significantly poor, with a reported median OS of 41 months.^15^ Another study showed that among a group of patients, the progression time from SMM to symptomatic MM was 28 months in patients with the t(4;14) compared with 55 months in patients with t(11;14).^16^ Other representative translocations include t(14;16) involving MAF, t(14;20) involving MAFB, and t(6;14) involving CCND3 (Table 1).^17^, ^18^, ^19^, ^20^, ^21^ Another possible driving event is aneuploidy, including hypodiploidy, pseudodiploidy, and the most frequent entity, hyperdiploidy. Chretien et al. conducted a genomic analysis by single‐nucleotide polymorphism array, and a cohort of 965 patients was enrolled. At least one trisomy was found in 61% of patients, with chromosome 9, 15, 19, 5, 3, 11, 7, 21, 18, or 17 trisomy. Most of the trisomies were associated with a protective effect on survival, except trisomy 17, 18, and 21. The patients with trisomy 3 showed a significantly longer time interval between diagnosis and progression than patients without a trisomy of chromosome 3. And patients who had a trisomy 21 showed worse outcomes than patients lacking this trisomy. Besides, patients with hypodiploidy were related to shorter time to progression and shorter OS.^22^

**TABLE 1:** Primary chromosomal translocations related to 14q32

Cytogenetic abnormality	Affected genes	Approximate frequency (%)	Refs.
t(11;14)	CCND1	14–21	11, 13, 15
t(4;14)	NSD2, FGFR3	10–15	11, 13, 15
t(14;16)	MAF	3–5	11, 19, 20
t(6;14)	CCND3	1–4	11, 13, 18
t(14;20)	MAFB	1–2	11, 14, 21

The short arm deletion of chromosome 17 (del(17p)) is one of the major abnormalities that impair the survival of patients. The median OS was 22 months in a group of transplant‐eligible patients with del(17p).^15^ In addition, patients with acquired del(17p) after treatment showed shorter median progression‐free survival (PFS) and OS compared with the control group without acquiring del(17p) at a comparable time point. The median PFS and OS were 5.4 and 18.1 months, respectively, after the detection of del(17p).^23^ Recently, scholars conducted a study on a large group of patients with del(17p) presenting in more than 55% of their plasma cells. Next‐generation sequencing targeting on TP53 was performed after homogeneous treatment. The results indicated that a group of patients showed the worst survival with the situation when del(17p) was associated with TP53 mutation. Nevertheless, del(17p) alone was also a very high‐risk feature associated with a poor outcome compared with the control cohort lacking del(17p).^24^ Other chromosomal abnormalities observed in MM patients include loss of the short arm of chromosome 1 (del(1p)), deletion of the long arm of chromosome 13 (del(13q)), and gain of the long arm of chromosome 1 (gain(1q)).^25^ The amplification of 1q21 exists in a certain portion of MM patients, and this unfavorable cytogenetic abnormality has been associated with poor response to standard treatment.^26^

The frequency of somatic mutations varies among patients. One study found that KRAS and NRAS mutated exclusively in MM patients, 21.2 and 19.4% in 463 patients, respectively.^27^ Other frequently mutated genes include BRAF, FAM46C, and DIS3. Besides, TRAF3, CYLD, RB1, IRF4, EGR1, and MAX are recurrently mutated genes.^28^ Structural variants involving MYC oncogene are common in MM patients. Deregulated MYC expression can promote genome instability and is associated with chromosomal rearrangements, leading to progression from newly diagnosed MM (NDMM) to a refractory state.^29^ In addition, epigenetic alterations also play an important role in myeloma. The global DNA methylation levels vary among patients, while levels of hypomethylation are increased in MM compared to precursor stages.^4^

### 2.2 Related signaling pathways

These genetic abnormalities affect several signaling pathways. The nuclear factor kappa B (NF‐κB) pathway, mitogen‐activated protein kinase (MAPK) pathway, and cell cycle pathway have been involved. The NF‐κB pathway is a pivotal signaling pathway for the development of lymphocytes, and lymphoid malignancies are always associated with the dysregulation of this pathway. Increased NF‐κB activity can be caused by gene mutations. Positive regulators, such as NF‐κB‐inducing kinase (NIK) and upstream receptor CD40, were overexpressed in MM.^30^ Other genetic abnormalities include mutations in NF‐κB2, and loss of function of negative regulators such as TRAF2, TRAF3, and CYLD.^11^ Activation of the NF‐κB pathway is mostly via two ways. In the canonical pathway, the activated IκB kinase (IKK) complex phosphorylates IκB proteins, resulting in the accumulation of heterodimers p50/p65 and c‐Rel/p65 in the nucleus. Unlike other B‐cell malignancies, MM is mostly related to the noncanonical NF‐κB pathway. NIK is necessary for the noncanonical pathway and is activated after initial stimulation. IKK‐α homodimers phosphorylate P100, which results in removal of the C‐terminal domain and accumulation of the p52/Rel‐B heterodimers in the nucleus.^31^ The proteins of the NF‐κB family are essential for nuclear translocation and DNA binding, and aberration of the NF‐κB pathway contributes to the initiation and progression of MM by regulating the expression of several genes associated with the growth, survival, and angiogenesis of MM.^32^

The MAPK pathway is a fundamental mediator of many biological processes involved in cell proliferation, growth, adhesion, and apoptosis. Genetic alterations in MM are associated with pathway activation. For instance, the t(4; 14) translocation leads to overexpression of FGFR3 and then stimulates the signaling cascade. RAS mutations, generally represented by NRAS and KRAS, are associated with the aberration of this pathway in MM.^27^ These mutations that activate MAPK signaling have been evidenced in aggressive myeloma cases. In particular, RAS mutation has been found in more advanced clinical scenarios with a shorter time to progression.^33^ RAS activates downstream targets and promotes the recruitment and phosphorylation of RAF, which then phosphorylates MEK. And MEK phosphorylates ERK in turn.^34^ Activated ERK can phosphorylate numerous downstream targets in the nucleus and cytoplasm. Moreover, RAS mutations may enhance proteasome capacity and reduce cellular stress, thereby inhibiting the efficacy of proteasome inhibitor (PI) and correlating with PI resistance.^35^ A preclinical study proved that with the inhibitory effect of sorafenib, which targets RAF and VEGFR2, apoptosis was induced among a panel of MM cell lines and in vivo models.^36^ Increased genome instability, both initiating and secondary genetic events, leads to cell cycle dysregulation in MM. Representative abnormalities include the overexpression of CCND1, CCND2, CCND3, and CKS1B, mutations of TP53 and RB1, leading to cell proliferation and clonal growth.^37^ In addition, dysregulation of the apoptotic pathway occurs, with increased expression of antiapoptotic proteins.

### 2.3 Bone marrow microenvironment

The correlation between MM cells and the BM microenvironment is associated with the pathogenesis of MM, contributing to the activation of signaling pathways and participating in the survival, progression, migration, and drug resistance of MM cells. Both cellular and noncellular components of BM niches play a role in the generation of tumor progression microenvironment. BM stromal cells (BMSCs) play a vital role in MM cell growth. The CXCL12 expressed on the surface of BMSCs binds to CXCR4 expressed on MM cells, mediating the homing and retention of MM cells in the BM. Furthermore, very late antigen‐4 integrin (VLA‐4) on MM cells binds to its ligand, vascular cell adhesion molecule 1, facilitating the trafficking of MM cells into BM niches.^38^ Other molecules, including α4β7 integrin, P‐selectin glycoprotein ligand‐1, and CD147, also contribute to cell adhesion and migration.^39^, ^40^

**FIGURE 3:**
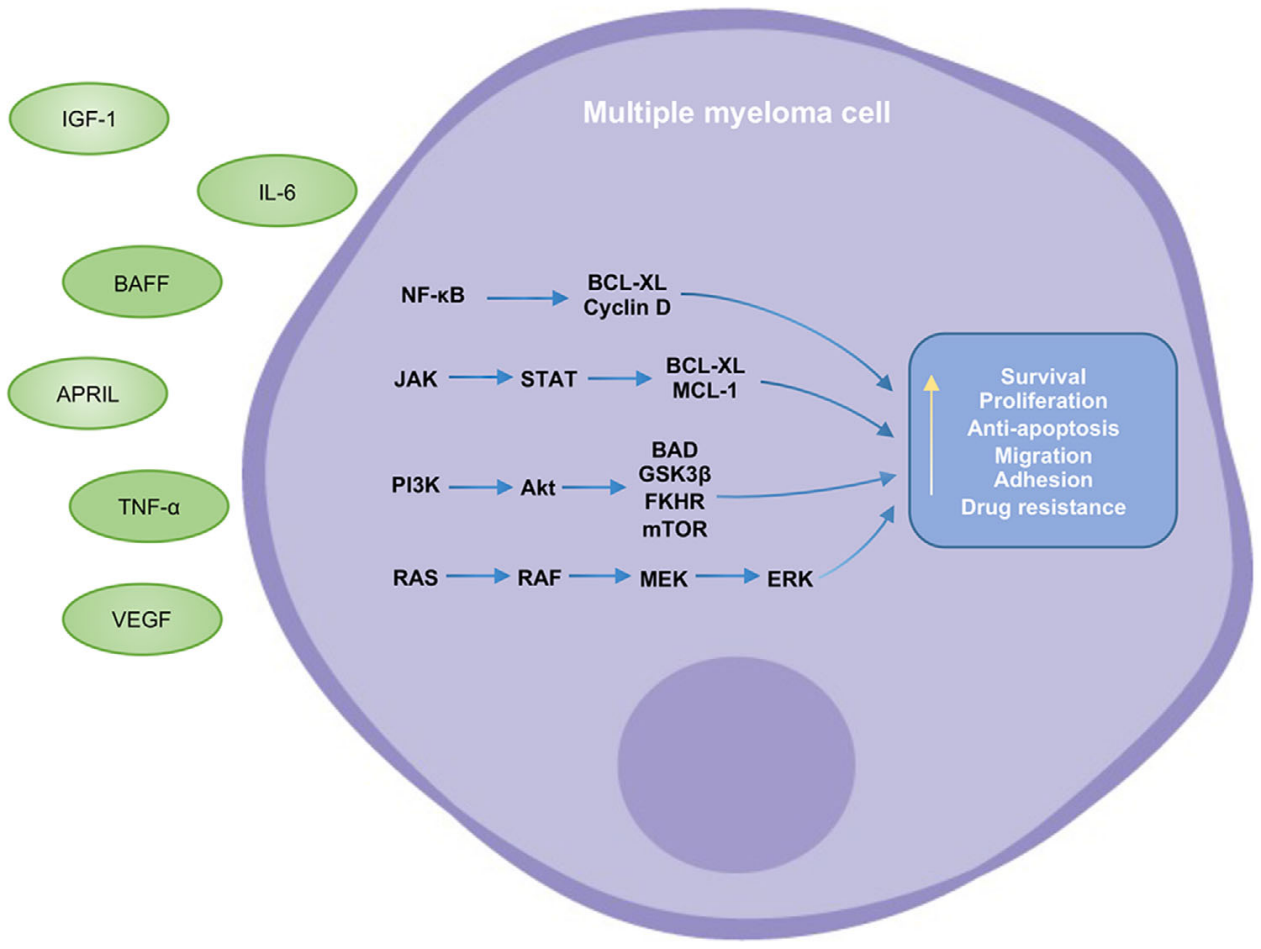
Roles of cytokines and signaling pathways in the pathogenesis of multiple myeloma. Cytokines and growth factors have been secreted in the bone marrow microenvironment such as IL‐6, IGF‐1, BAFF, APRIL, TNF‐α, and VEGF. These soluble factors activate signaling pathways, including the NF‐κB pathway, PI3K/Akt/mTOR pathway, JAK/STAT pathway, and MAPK pathway, which regulate the survival, proliferation, apoptosis, migration, adhesion, and drug resistance of myeloma cells

The interaction between MM cells and the BM microenvironment leads to considerable secretion of cytokines and growth factors, including IL‐6, insulin‐like growth factor (IGF‐1), B‐cell activating factor (BAFF), a proliferation‐inducing ligand (APRIL), tumor necrosis factor‐α (TNF‐α), and vascular endothelial growth factor (VEGF).^41^ These soluble factors activate intracellular signals that regulate the growth, proliferation, migration, and drug resistance of malignant cells. The secretion of BAFF, APRIL, and TNF‐α stimulates the NF‐κB signaling pathway.^32^ Besides, IGF‐1 has been reported to activate the NF‐κB pathway indirectly and is needed for the survival of MM cells.^42^ IL‐6 is another growth factor for MM cells and is involved in the NF‐κB pathway. Activation of the NF‐κB signal can also promote the production of several factors, including IL‐6, BAFF, and APRIL, thus, resulting in a positive feedback loop that allows for constitutive activation of NF‐κB and, consequently, the augmented survival and proliferation of MM cells.^31^ In addition to the NF‐κB pathway, PI3K/Akt/mTOR signaling pathway, JAK/STAT pathway, and MAPK pathway have also been involved in this response. Downstream sequelae include dysregulation of cytokines, cell‐cycle regulatory proteins, and antiapoptotic proteins (Figure 3).^43^, ^44^

Drug resistance is a crucial issue in tumor treatment. The binding of MM cells to BMSCs causes cell adhesion‐mediated drug resistance. Endothelial cells, macrophages, stromal cells, and other components in the BM microenvironment are involved in the formation of vascular niches that promote the proliferation and survival of MM cells, and protect them from antimyeloma agents.^45^ In addition, factors in the microenvironment, such as the VEGF family, monocyte chemotactic protein‐1 (MCP‐1), TNF‐α, and IL‐8, are important for enhanced angiogenesis in MM, which is parallel with disease progression.^45^ The BM microenvironment can mediate immune escape via the immune suppression of regulatory T cells, regulatory B cells, and myeloid‐derived suppressor cells (MDSCs). MDSCs can suppress immune responses, especially driven by the activation of the STAT3 pathway, and inhibit the proliferation of cytotoxic T lymphocytes and nature killer (NK) cells.^46^ Besides, osteoclasts play a role in the immunosuppressive microenvironment. With the stimulation of signaling pathways, the BM microenvironment acquires antiapoptotic effects via the increase of antiapoptotic regulatory proteins.^47^ For instance, BCL‐2, BCL‐XL, and MCL‐1 have been significantly upregulated. Increased soluble factors and surface molecules, such as IL‐6, transforming growth factor‐β (TGF‐β), IL‐10, APRIL, ICAM‐1, and CD40, also contribute to immune escape and resistance against immune effector cells.

Exosomes establish cell–cell communication and act in the interplay between the BM microenvironment and MM cells. The content inside exosomes has been identified as different between MM patients and healthy donors, which can serve as tumor biomarkers and therapeutic targets.^48^ BMSC‐derived exosomes were loaded with higher levels of oncogenic proteins, cytokines, and adhesion molecules, including IL‐6 and fibronectin, while expressed a lower level of tumor‐suppressive factors, promoting cell growth and facilitating MM progression.^49^ MM‐derived exosomes were enriched with amphiregulin, which led to the activation of the epidermal growth factor pathway in preosteoclasts and osteoclastogenesis.^50^ Furthermore, exosomes in the BM microenvironment have been shown to be related to drug resistance, enhanced angiogenesis, and the generation of the immunosuppressive environment.^51^, ^52^

### 2.4 Myeloma‐related bone disease

**FIGURE 4:**
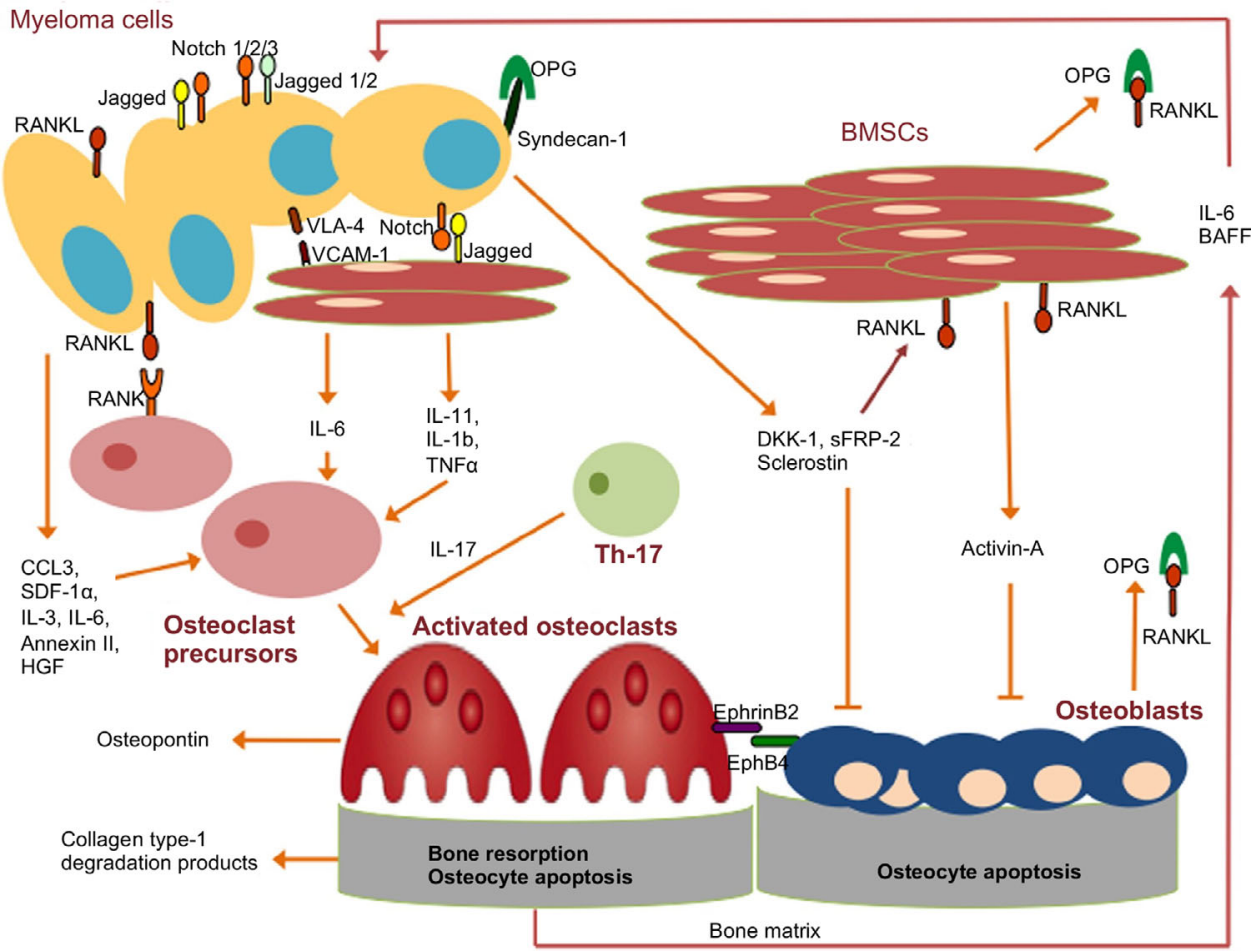
Disturbance of the balance between the bone‐repairing osteoblasts and bone‐resorbing osteoclasts leads to myeloma‐related bone disease. The interaction between the bone marrow microenvironment and MM cells induces the release of cytokines and pro‐osteoclastogenic factors, increasing osteoclast activity and inhibiting osteoblastogenesis (Copyright 2018, Springer Nature^53^)

The osteolytic bone disease is one of the symbolized manifestations of MM. The aberrant bone formation process is caused by disturbance of the balance between the bone‐repairing osteoblasts and bone‐resorbing osteoclasts (Figure 4).^53^ Through the cell contact between MM cells and BMSCs, high levels of osteoclastogenic stimuli have been produced, mainly including receptor activator of NF‐κB ligand (RANKL). RANKL binds to the receptor activator of NF‐κB (RANK) on the surface of osteoclasts, stimulating their differentiation and promoting their activity. Osteoprotegerin (OPG) performs as a decoy RANK receptor to prevent the binding of RANKL and RANK, inhibiting the process of osteoclastogenesis.^8^ However, the interaction between MM cells with BMSCs and osteoblasts can decrease the level of OPG and increase the expression of RANKL. The serum level of soluble RANKL/OPG ratio is associated with bone resorption and osteolytic lesions.^54^ Macrophage inflammatory protein‐1α (MIP‐1α) is an osteoclast stimulator secreted by MM cells and is related to bone destruction in MM. MIP‐1α interacts with its receptor and then induces the production of osteoclasts.^55^ Besides, TNF‐α produced by MM cells is associated with the activation of a number of signaling pathways, not only enhancing the growth of MM cells, but also promoting the differentiation of osteoclasts.^56^

In contrast to the increased activity of osteoclasts, differentiation and activity of osteoblasts have been severely impaired and then fail to repair bone destruction. The Wnt/β‐catenin cascade is a conserved signaling pathway and plays a dominant role in regulating the proliferation, survival, and differentiation of osteoblasts, responsible for the balance between bone forming and bone resorbing.^57^ The secretion of Wnt antagonists can disturb the balance in the BM and lead to the development of osteolytic bone lesions. A high level of dickkopf‐1 (DKK‐1), the canonical Wnt inhibitor, has been found to be related to decreased bone formation and the presence of bone lesions in animal models and patient samples.^58^ In addition, Wnt signaling also plays a more indirect role in influencing the RANK/RANKL/OPG signaling axis. Other factors, such as sFRP‐2, sclerostin, and activin‐A, impede the production of osteoblasts. Releasing of TGF‐β, IL‐17, IL‐3, and IL‐6 also collaborates to impair the balance of bone formation.^59^ It is conceivable that through these intensive studies, the pathogenesis and molecular mechanisms have been deciphered, thus, providing more strategies for the treatment of MM.

## 3 CURRENT TREATMENTS FOR MULTIPLE MYELOMA

With the presence of PIs, immunomodulatory drugs (IMiDs), monoclonal antibodies (mAbs), and novel therapies, the PFS and OS of MM have been significantly improved in recent decades.^4^ MM has a great variety of genetic abnormalities, and there is a crosstalk between intracellular signaling pathways, making it a complicated network. The primary goals of MM treatment are to control disease progression, suppress malignancy over the long term, mitigate disease‐related complications, and increase survival. Combination therapies are effective strategies to achieve favorable outcomes. The treatment process usually includes induction, consolidation, and maintenance therapy. The eligibility for autologous stem cell transplantation (ASCT) should be evaluated according to age, comorbidities, and risk stratification of patients. The triplet therapy regimen, RVd (lenalidomide, bortezomib [BTZ], and dexamethasone), is currently a standard for initial treatment.^60^ And the quadruplet regimen with added daratumumab has gained success in high‐risk patients, followed by ASCT and lenalidomide maintenance therapy.^61^ Relapse is inevitable in many MM patients. For the purpose of long‐term disease control, new generation PIs and IMiDs, including ixazomib and pomalidomide, could be preferred options for relapsed patients.^62^ Besides, isatuximab‐containing regimens have shown promising outcomes.^63^ Selinexor, panobinostat, and belantamab mafodotin have been approved for second or higher relapse. And chimeric antigen receptor (CAR) T‐cell therapy has been evidenced to have unprecedented efficacy in relapsed or refractory MM (RRMM) patients as well. The optimal choice of agents can be decided depending on several factors including patient features, previous treatments, and disease characteristics. The primary treatment options and representative regimens are summarized in Table 2.

**TABLE 2:** Pharmacological options for multiple myeloma

Categories	Agents	Mechanisms	Representative regimen	ClinicalTrials.gov number
Proteasome Inhibitor	Bortezomib	Inhibition of 20S proteasome; increased stress of the endoplasmic reticulum; inhibition of the NF‐κB pathway	Bortezomib, lenalidomide, and dexamethasone	NCT00644228
	Carfilzomib		Carfilzomib, lenalidomide, and dexamethasone	NCT01863550
	Ixazomib		Ixazomib, lenalidomide, and dexamethasone	NCT01850524
	Marizomib		Marizomib and dexamethasone	NCT00461045
	Oprozomib		Oprozomib and dexamethasone	NCT01832727
Immunomodulatory drug	Thalidomide	Targets cereblon; immunomodulatory effect	Thalidomide, bortezomib, and dexamethasone	NCT01134484
	Lenalidomide		Bortezomib, lenalidomide, and dexamethasone	NCT00644228
	Pomalidomide		Pomalidomide, bortezomib, and dexamethasone	NCT01734928
Monoclonal antibody	Daratumumab	Anti‐CD38 monoclonal antibody	Daratumumab, bortezomib, and dexamethasone	NCT02136134
	Isatuximab		Isatuximab, pomalidomide, and dexamethasone	NCT02990338
	MOR202		MOR202, pomalidomide/lenalidomide, and dexamethasone	NCT01421186
	Elotuzumab	Anti‐SLAMF7 monoclonal antibody	Elotuzumab, pomalidomide, and dexamethasone	NCT02654132
	BHQ880	Anti‐DKK‐1 monoclonal antibody	BHQ880, zoledronic acid, and anti‐myeloma therapy	NCT00741377
CAR T‐cell therapy	Idecabtagene vicleucel	CAR T‐cell therapy targets BCMA	Monotherapy	NCT02658929
	Ciltacabtagene autoleucel		Monotherapy	NCT03548207
Small‐molecule inhibitor	Panobinostat	Inhibition of pan‐deacetylase	Panobinostat, bortezomib, and dexamethasone	NCT01023308
	Ricolinostat	Selectively inhibition of histone deacetylase 6	Ricolinostat, bortezomib, and dexamethasone	NCT01323751
	Venetoclax	Inhibition of BCL‐2	Venetoclax, dexamethasone	NCT01794520
	Selinexor	Inhibition of exportin 1	Selinexor, dexamethasone	NCT02336815
	Filanesib	Inhibition of kinesin spindle	Filanesib, pomalidomide, and dexamethasone	NCT02384083
Other novel therapies	Belantamab mafodotin	Antibody‐drug conjugate targets BCMA	Monotherapy	NCT03525678
	AMG 420	Bispecific T‐cell engager targets BCMA and CD3	Monotherapy	NCT02514239
	Teclistamab	Bispecific antibody binds to BCMA and CD3	Monotherapy	NCT03145181

### 3.1 Pharmacological therapy

#### 3.1.1 Proteasome inhibitors

The proteasome regulates protein catabolism through the ubiquitin‐proteasome pathway. The 20S proteasome core is the vital catalytic site for degrading deubiquitinated proteins and releasing oligopeptides.^64^ PIs are one of the most promising agents for NDMM patients and RRMM patients, and several mechanisms of action have been demonstrated. When the proteasome function is inhibited, proteins inside cells accumulate in the cytoplasm, leading to the increased stress of the endoplasmic reticulum, disrupting cell‐cycle signals, and activating apoptotic pathways.^65^ Another mechanism is the inhibition of NF‐κB activity. PIs can inhibit the degradation of the inhibitor of NF‐κB, thus, blocking the progression of the disease.^32^

BTZ is a first‐in‐class PI that has been approved by the FDA since 2003.^66^ BTZ‐based regimens have been the first‐line and cornerstone option for MM. BTZ leads to apoptosis directly, inactivates the NF‐κB pathway, inhibits the production of IL‐6 and IGF‐1, and prevents the adherence of myeloma cells to the BM microenvironment.^67^ In clinical trials, BTZ‐based regimens have achieved a high overall response rate (ORR), and prolonged PFS and OS.^68^ A phase 3 clinical trial recruited patients at 139 institutions and randomized them into the RVd group or the Rd (lenalidomide and dexamethasone) group. The median PFS was meaningfully longer in the RVd group than that in the Rd group. And the median OS was also greatly improved in the RVd group.^69^ After longer follow‐up, the updated report included 460 patients for survival endpoints analysis. The PFS in RVd group was 41 months, while in the Rd group, it was 29 months.^70^ The RVd regimen results in a clinically meaningful improvement and occupies a vital position in treatment approaches for MM. Adverse effects of BTZ include fatigue, gastrointestinal symptoms, thrombocytopenia, and peripheral neuropathy. A study compared the efficacy of subcutaneous and intravenous BTZ in induction therapy for NDMM patients. After long‐term follow‐up, subcutaneous BTZ was not inferior to intravenous BTZ. Moreover, the occurrence of nervous system disorders during maintenance therapy decreased in patients treated with subcutaneous BTZ.^71^

Carfilzomib is a second‐generation PI indicated for MM patients who exhibit invalidation or relapse after receiving at least two kinds of drugs.^72^ The mechanism of carfilzomib is different from that of BTZ, which binds to the chymotrypsin‐like proteasome site irreversibly and exclusively, leading to apoptosis consequently.^73^ The single‐agent clinical trial of carfilzomib showed that the ORR was 23.7% in 257 heavily pretreated patients. And the median OS was 15.6 months.^74^ Adding carfilzomib with lenalidomide and dexamethasone (KRd) demonstrated superior benefits over the control group. The PFS in the carfilzomib group was 26.3 months compared to 17.6 months in the control group.^75^ Carfilzomib has notably extended the PFS while the rate of adverse events was slightly higher. These results indicated durable responses of carfilzomib in RRMM patients. A large group of NDMM patients were randomized to receive either the RVd regimen or the KRd regimen. The median PFS was 34.6 months in the KRd group and 34.4 months in the RVd group. The KRd group did not show a better outcome in NDMM patients, and the treatment‐related adverse events occurred more frequently in the KRd group.^76^ In addition, the safety profile of carfilzomib was analyzed from four phase 2 trials. The most common grade 3 or higher adverse events were thrombocytopenia, anemia, lymphopenia, and pneumonia. Aggregated cardiac failure events were reported in 7.2% of patients. Overall, the results indicated a tolerable safety profile of carfilzomib in patients.^77^

Ixazomib is an orally administrated, reversible PI that acts by inhibiting the chymotrypsin‐like activity of the β5 subunit of the 20S proteasome.^78^ In a phase 3 randomized clinical trial, the ORR was 78.3% in the IRd (ixazomib, lenalidomide, and dexamethasone) group compared with 72% in the placebo‐Rd group in RRMM patients. The median duration of response and PFS in the IRd group was longer than that in the placebo‐Rd group. Meanwhile, the IRd group showed a substantial benefit in high‐risk patients. Regarding safety, adverse events of grade 3 and higher happened in 74 and 69% of the patients in each group.^79^, ^80^ Nevertheless, after longer follow‐up, there was no statistically significant benefit of OS in the IRd group. In a clinical trial that recruited transplant‐ineligible NDMM patients, the median PFS was 35.3 months in the IRd group and 21.8 months in the placebo‐Rd group, with primarily tolerable safety profiles.^81^ This oral combination regimen can be a feasible choice for these patients. Besides, some other compounds are underway. Marizomib is an irreversible PI, and a phase I trial demonstrated its safety and activity in RRMM patients.^82^ Oprozomib is orally administrated, and antimyeloma activity has been identified in NDMM patients while accompanied by gastrointestinal toxicities.^83^

#### 3.1.2 Immunomodulatory drugs

The IMiDs have been reported to target the cereblon ubiquitin ligase and lead to the degradation of Ikaros family zinc finger proteins 1(IKZF1) and 3 (IKZF3), thus, downregulating the downstream targets including the expression of IRF4.^84^ Besides, IMiDs have been evidenced to reduce the production of IL‐6 and IL‐16, increase IL‐2, interfere with the interaction between BMSCs and MM cells, and exhibit antitumor and immunomodulatory effects.^85^, ^86^ Thalidomide is the first IMiD used in MM, and now this drug is still a member of several regimens combined with BTZ, melphalan, and dexamethasone. In a randomized phase 3 study, patients received thalidomide and dexamethasone (Td) regimen, either alone or with BTZ (VTd), followed by ASCT and VTd or Td consolidation therapy. The 10‐year‐PFS was 34% in the VTd group and 17% in the Td group.^87^ The second‐generation IMiD, lenalidomide, has a potent immunomodulatory effect in the costimulation of T cells.^88^ This drug was initially approved for RRMM combined with dexamethasone. A complete or partial response in the lenalidomide group was 60.2%, which was significantly better than that in the placebo group.^89^ Thereafter, the carfilzomib, lenalidomide, and dexamethasone regimen were evidenced to be tolerable and highly effective, demonstrating favorable rates of minimal residual disease (MRD) negativity in NDMM.^90^ Currently, lenalidomide‐based regimens are fundamental and valuable for MM. The combination of lenalidomide and several PIs, mAbs, or other novel agents has been explored in clinical trials.^81^, ^91^

For lenalidomide‐refractory patients, pomalidomide is a suitable option. Pomalidomide can mitigate lytic bone disease, and the anti‐inflammatory effect has also been demonstrated.^92^ Pomalidomide with low‐dose dexamethasone is efficacious for myeloma patients who have received more than two prior therapies. The median PFS and OS were 4.2 months and 16.5 months, respectively, and ORR was 33%.^93^ Scholars assessed the safety and efficacy of cyclophosphamide, pomalidomide, and dexamethasone regimen. The median PFS was 9.5 months, and the ORR was 64.7% in this triplet group. This combination therapy showed a superior efficacy to the pomalidomide and dexamethasone regimen.^94^ Another triplet regimen containing pomalidomide, BTZ, and dexamethasone (PVd) has been investigated. The ORR among all evaluable patients was 86%, with a stringent complete response of 12%, and the median PFS was 13.7 months.^95^ In the OPTIMISMM trial, which was a randomized phase 3 multicentre clinical trial, the median PFS was 11.2 months in lenalidomide‐refractory patients when treated with PVd regimen, compared with 7.1 months in the BTZ and dexamethasone group.^96^ This result indicated that the PVd combination therapy is a manageable and efficacious option for lenalidomide‐refractory patients. Since the synergistic efficacy between PIs and IMiDs, pomalidomide has been studied in combination with carfilzomib and dexamethasone. And the combination therapy showed acceptable safety profiles.^97^ In addition, once‐weekly carfilzomib, pomalidomide, and dexamethasone were assessed. The median PFS was 10.3 months, and the median OS was not reached, with a median follow‐up of 12.8 months. Any grade adverse events included neutropenia, thrombocytopenia, anemia, infections, and vascular events.^98^

#### 3.1.3 Monoclonal antibodies

CD38 is a glycoprotein that acts as an extracellular enzyme.^99^ It is related to cell adhesion and cytokine secretion, and is expressed highly and coincidently on MM cells, making it a potential target.^100^ Daratumumab is an immunoglobulin G1 kappa mAb that binds to CD38.^101^ Daratumumab monotherapy has exhibited encouraging efficacy in heavily pretreated patients. The ORR was 29.2%, and the median PFS was 3.7 months.^102^ A study performed a pooled analysis of two daratumumab monotherapy trials, in which 86.5% of patients were double refractory to a PI and an IMiD. This pooled analysis indicated a clinical benefit with deep and durable responses of daratumumab monotherapy. Common adverse events included fatigue, upper respiratory tract infection, nausea, anemia, back pain, and thrombocytopenia. The infusion‐related reactions (IRRs) occurred in 48% of patients, and mostly during the first infusion.^103^ In addition, daratumumab has shown decisive action when combined with BTZ and dexamethasone.^104^ The PFS in the Dara‐Vd (daratumumab, BTZ, and dexamethasone) group was 16.7 months, while it was only 7.1 months in the BTZ and dexamethasone group. Even in the high cytogenetic risk subgroup, the PFS was greatly prolonged in the Dara‐Vd group. Besides, the rate of MRD negativity was also significantly improved, demonstrating favorable clinical benefit.^105^, ^106^ The POLLUX trial evaluated another triplet regimen, Dara‐Rd (daratumumab, lenalidomide, and dexamethasone). The PFS was 44.5 months in the Dara‐Rd group compared to 17.5 months in the control group, and the ORR in the Dara‐Rd group was 92.9%, accompanied with deep responses.^107^ To relieve IRRs, subcutaneous infusion of daratumumab has been applied, with recombinant human hyaluronidase enzyme. A total of 522 patients were recruited in the COLUMAB trial, and the ORR was 41% in the subcutaneous group compared with 37% in the intravenous group, indicating that the subcutaneous formulation was noninferior to the intravenous formulation with an improved safety profile.^108^ In addition, the four‐drug regimen incorporating daratumumab, BTZ, lenalidomide, and dexamethasone has been adopted for transplantation‐eligible NDMM patients, aiming for more profound disease responses.^91^ These inspiring results support the status of daratumumab‐based regimens for both RRMM and NDMM patients.

Isatuximab is another antibody that binds to CD38 and targets an amino acid sequence different from that of daratumumab.^109^ Isatuximab monotherapy showed an ORR of 23.9% in RRMM patients, and when combined with dexamethasone, the ORR was 43.6%.^110^ A multicentre phase 3 clinical trial recruited 307 patients to determine the PFS benefit of isatuximab, pomalidomide, and dexamethasone combinational therapy. This triplet regimen significantly improved the PFS, with a median PFS of 11.5 months.^63^ IRRs, upper respiratory tract infections, and diarrhea were the most common adverse events. The combination of isatuximab and PIs has also been assessed. The addition of isatuximab to carfilzomib and dexamethasone regimen showed improved PFS and depth of response in RRMM patients.^111^ Isatuximab has become an important new treatment option for the management of this population.

Elotuzumab binds to signaling lymphocytic activation molecule family member 7 (SLAMF7), which is expressed in NK cells and myeloma cells.^112^ Elotuzumab has been shown to eliminate myeloma cells by downregulating target expression and inducing NK cell‐mediated antibody‐dependent cell cytotoxicity.^113^ The combination of elotuzumab and lenalidomide was reported to enhance NK cell function and myeloma cell killing efficacy, with increased secretion of IL‐2 and production of TNF‐α.^114^ In a clinical trial, the elotuzumab, pomalidomide, and dexamethasone regimen showed prolonged PFS.^115^ Furthermore, aberration of the Wnt/β‐catenin cascade has been involved in the progression of MM. BHQ880 is a DKK‐1 neutralizing antibody, and Fulciniti et al. reported that BHQ880 could increase osteoblast differentiation and decrease IL‐6 secretion.^116^ BHQ880 combined with antimyeloma therapy and zoledronic acid was tolerable and demonstrated potential activity in RRMM patients.^117^

#### 3.1.4 Chimeric antigen receptor T‐cell therapies

CAR T‐cell therapy possesses a high status and revolutionizes the treatment of hematological malignancies. Autologous T cells adopted from MM patients have been engineered with CARs to recognize specific antigens and then transfused back into patients.^118^ The CARs usually consist of an antigen‐binding domain, followed by a hinge and transmembrane domain. The signaling domains include costimulatory domain and activation domain, which are essential for activating cytokine production and cytolytic capacity.^119^ In addition, choosing a proper target is vital, and B‐cell maturation antigen (BCMA), CD38, and CD19 are attractive targets in MM.^120^, ^121^ A group of patients who received salvageable therapy and ASCT were injected with anti‐CD19 CAR T‐cells, and 2 of 10 patients showed significantly longer PFS.^122^ BCMA belongs to the TNF receptor superfamily.^123^ Besides, BCMA is significantly expressed in malignant cells and a limited portion of nonmalignant cells, including plasma cells and a small subset of B cells, leading to activation of the NF‐κB pathway, and multiple‐step progression in MM patients.^124^ Idecabtagene vicleucel (Bb2121) is the first anti‐BCMA CAR T‐cell product approved by the FDA in 2021, which consists of an anti‐BCMA single‐chain variable fragment, a 4‐1BB costimulatory motif, and a CD3‐zeta signaling domain. In a phase 1 trial, the ORR was 85%, and 45% of patients achieved a complete response. Hematologic toxic effects were common events, and cytokine release syndrome occurred in 76% of patients.^125^ In a phase 2 study, 128 patients received Bb2121 with a median follow‐up over 1 year. The ORR was 73%, and 33% of patients had a complete or better response. Besides, the median PFS was 8.8 months, and 33 of 128 patients achieved MRD‐negative status. Toxic effects occurred in almost all patients, and the rate of cytokine release syndrome was 84%.^126^ In addition to Bb2121, the second anti‐BCMA CAR T‐cell product, Ciltacabtagene autoleucel, has been approved recently, and plenty of CAR T‐cell therapies targeting BCMA or other targets are in active development.^127^

#### 3.1.5 Small‐molecule inhibitors

Some subgroups of MM patients, including frail elderly, high‐risk, and refractory patients, remain suboptimal in survival outcomes. And novel agents are needed to improve the prognosis further. The histone deacetylase inhibitors (HDACis), such as panobinostat and ricolinostat, have emerged as important choices.^128^ Histone deacetylase has been evidenced to be overexpressed in neoplasm cells and associated with poor prognosis.^129^ Furthermore, HDACis have exhibited the ability to induce differentiation and inhibit migration, invasion, and tumor growth in both animal models and patients.^130^ Panobinostat is an oral pan‐deacetylase inhibitor, the panobinostat, BTZ, and dexamethasone regimen was compared with the BTZ and dexamethasone regimen. The panobinostat group showed a longer PFS and a higher complete or near complete response rate. Nevertheless, serious adverse events were reported more in the panobinostat group, and common adverse events included thrombocytopenia, lymphopenia, diarrhea, asthenia or fatigue, and peripheral neuropathy.^131^ Another study combined oral panobinostat and dexamethasone with subcutaneous BTZ. The ORR in the panobinostat (20 mg), three‐times‐weekly group was 62.2%, and the result indicated a favorable safety profile.^132^ In addition, the panobinostat and carfilzomib combination therapy resulted as a practical steroid‐sparing choice for RRMM.^133^ Ricolinostat is an HDAC6‐selective inhibitor, and the ricolinostat, BTZ, and dexamethasone combination therapy was tolerable at a ricolinostat dose of 160 mg daily, suggesting an acceptable safety profile and efficacy as well.^134^

The BCL‐2 protein family regulates the apoptosis pathway, and the abnormal expression or dysfunction of the BCL‐2 protein family is associated with carcinogenesis and resistance to anticancer drugs.^135^ BCL‐2 and MCL‐1, which belong to the prosurvival BCL‐2‐like proteins, represent attractive targets.^136^ Venetoclax is a highly selective BCL‐2 inhibitor and has demonstrated impressive results in clinical trials by directly provoking apoptosis of tumor cells.^137^ In a multicentre study, the combination of venetoclax and dexamethasone demonstrated efficacy and safety in heavily pretreated t(11;14) RRMM patients, with an ORR of 60% in phase 1 and 48% in phase 2.^138^ The venetoclax, BTZ, and dexamethasone regimen showed a median PFS of 22.4 months, notably longer than that in the placebo group.^137^ However, the rate of adverse events was higher in the venetoclax group, with increased mortality, limiting this treatment option.

Exportin 1 (XPO1) is responsible for the nuclear export of more than 200 proteins, many of which are tumor suppressor proteins, and XPO1 is overexpressed in several tumor cells.^139^ Selinexor is the first XPO1 inhibitor approved by the FDA for RRMM.^140^ In triple‐class refractory patients, selinexor plus dexamethasone achieved a partial or better response of 26%, with two stringent complete responses. The median PFS and OS were 3.7 months and 8.6 months, respectively, demonstrating a favorable efficacy for these heavily pretreated and refractory patients.^141^ The combination of once‐per‐week selinexor with BTZ and dexamethasone was assessed. The median PFS was 13.9 months in this triplet regimen and 9.5 months in the BTZ and dexamethasone regimen. Despite that, adverse events occurred more frequently in the selinexor group including thrombocytopenia, anemia, fatigue, and pneumonia.^142^ Thereafter, patients treated with selinexor need supportive management properly. In addition, the combination of selinexor with carfilzomib is under exploration.^143^

#### 3.1.6 Other novel therapies

Immunotherapy has been a powerful strategy in tumor treatment. This approach may be the key to regaining immune balance and generating durable control of myeloma.^144^ In addition to mAbs and CAR T‐cell therapies, bispecific T‐cell engagers have been introduced into preclinical studies and clinical trials. AMG‐420 is a bispecific T‐cell engager that binds to BCMA on target cells, leading to T cell‐mediated lysis. The first‐in‐human study showed an ORR of 70% while requiring continuous infusions.^145^ Teclistamab is a humanized bispecific antibody that binds to BCMA and CD3, and demonstrated promising efficacy and durable responses in a phase 1 study.^146^ An antibody‐drug conjugate is the combination of a recombinant mAb with a cytotoxic chemodrug.^147^ It first binds to the target and then delivers the cytotoxic agent into myeloma cells. Belantamab mafodotin targeting BCMA showed 31 and 34% overall response in different dose cohorts in a two‐arm phase 2 study.^148^ The most common adverse events included anemia, keratopathy, and thrombocytopenia. As more agents are approved, more studies are needed to properly incorporate these strategies with present therapies, and reduce treatment‐related toxicities.

### 3.2 Autologous stem cell transplantation

Although numerous therapeutic agents have been introduced for the treatment of myeloma, ASCT is still a cornerstone in MM after initial treatment. Hematopoietic stem cells are mobilized into peripheral blood by granulocyte colony‐stimulating factors with or without chemotherapy. The CD34^+^ stem cells are collected at least 2 × 10^6^ cells/kg. After that, patients receive conditioning therapy, with high‐dose melphalan at the 200 mg/m^2^ level as the standard regimen. Other reduced dose levels, such as 140 mg/m^2^, are always considered for patients who are older than 65 and have worse renal function.^149^ A study reported that the at least very good partial response rate in the melphalan (200 mg/m^2^) group was higher than that in the lower dose group, and melphalan (200 mg/m^2^) was associated with a lower disease progression rate.^150^ Other clinical trials are now investigating combination regimens for conditioning therapy, for instance, busulfan in combination with melphalan.^151^ Debates on the role of ASCT still exist, and several clinical trials have demonstrated enhanced responses and survival benefits. Attal et al. reported that ASCT after the RVd combination therapy was associated with a higher complete response and a longer PFS than RVd therapy alone.^152^ Recently, a study pooled four randomized clinical trials for conventional meta‐analysis and five randomized clinical trials for network meta‐analysis. The results indicated that high‐dose melphalan, followed by ASCT was associated with superior PFS compared to standard‐dose therapy using novel agents, while there was no OS benefit.^153^ In addition, the optimal timing for ASCT and whether single or tandem ASCT still remains to be determined. Ongoing studies and long‐term follow‐up will continue to figure out a favorable profile to apply ASCT properly for patients with different risk profiles and performance status.

## 4 NANOMEDICINE‐BASED STRATEGIES FOR MULTIPLE MYELOMA

Despite the advances of effective managements, many MM patients eventually relapse and develop resistance to drugs.^154^ In addition to that, systemic toxicity and adverse events are obstacles of numerous regimens. Accordingly, nanomedicine‐based strategies are underway to address these potential issues and improve the treatment efficacy of MM.

Nanomedicine is the intentional design and application of nanoscale biomaterials in the field of medicine.^155^ Nanobiomaterials mainly include two broad categories, organic nanomaterials and inorganic nanomaterials.^156^ Liposomes, micelles, and polymeric nanoparticles have been widely applied as drug carriers; metal particles, graphene, and silicon dioxide nanoparticles can serve as both nanocarriers and diagnostic tools.^157^, ^158^, ^159^, ^160^ The physicochemical properties, such as composition, size, and surface charge, can influence the process of blood circulation, biodistribution, and cellular internalization in vivo. Tailoring the properties and responsive conditions of nanomedicine provides opportunities to achieve different goals. Encapsulating drugs into nanoparticle platforms can enhance the solubility and stability, control drug release, increase drug concentration at the tumor site, and reduce side‐effects at the same time.^161^, ^162^

The enhanced permeability and retention (EPR) effect contributes to the upgraded efficacy of nanomedicine in solid tumors, owing to the abundant generation of blood vessels and defective vascular structure inside and around neoplasms.^163^ However, the EPR effect varied among different types of tumors because of significant heterogeneity. Tumor type, size, location, surrounding environment, and mononuclear phagocytic system are critical factors correlated with the EPR effect.^164^ Meanwhile, pressure inside solid tumors and other biological barriers impedes the deep penetration of nanomedicine to some extent. In comparison, hematological malignancies benefit little from the EPR property.^165^ The long circulation property contributes to the retention of nanomedicine in the blood and BM, thus, facilitating the interaction of nanomedicine with hematological tumor cells.^166^ The increased angiogenesis and abundant blood flow in the BM contribute to the augmented passive accumulation. Furthermore, active targeting nanomedicine performs better among solid tumors and hematological tumors. Targeting strategies are established according to the different properties and molecular expression levels of tumors. Some specific molecules, such as peptides, antibodies, aptamers, and proteins, can be used as high‐affinity targeting agents or ligands to achieve precise treatment.^167^, ^168^ For MM, multidrug combination therapies have been broadly adopted, and codelivery of two or more drugs in one nanoparticle platform guarantees the synergistic drug ratios. In addition, nanotechnology also assists in immunotherapy and refines these strategies.^169^ In this section, we summarize the development and recent advances in nanomedicine‐based strategies for MM, elaborating the nanoparticle design and antitumor potency.

### 4.1 Liposomes

The liposome is one of the ideal and widely applied nanomaterials for drug delivery. Liposomes are composed of phospholipids and cholesterol. The aqueous core is capable of loading hydrophilic drugs, and the lipid bilayer can encapsulate hydrophobic drugs. Pegylated liposomal doxorubicin (PLD) was introduced into the market in 1995 for the treatment of MM and other tumors.^170^ To verify the efficacy of PLD, several clinical trials have been carried out. PLD reduced systemic toxicity, including cardiotoxicity, by long blood circulation and passive accumulation in the tumor sites.^171^ Dexamethasone occupies an essential position in standard regimens for MM. In an advanced human‐mouse hybrid MM model, the liposomal dexamethasone showed prolonged circulation and strong tumor inhibition, while free dexamethasone was ineffective at the same dosage.^172^ Other chemodrugs, such as gemcitabine, have also been loaded by pegylated unilamellar liposomes and showed increased apoptosis induction and cell proliferation inhibition compared to the free drug.^173^

To synthesize the BTZ prodrug, Ashley et al. mixed the pegylated lipids and BTZ‐conjugated lipids to obtain liposomal BTZ with a reversible boronic ester linkage. This liposomal BTZ exhibited upgraded pharmacokinetics and better efficacy in tumor growth inhibition.^174^ Several factors and preparation steps are essential for the design of liposomes. Entrapping agents, temperature, incubation duration, total lipid ratio, and drug concentration may change the properties. The diameter of liposomal carfilzomib was approximately 70 nm. Compared to free carfilzomib, the liposomal carfilzomib displayed improved effectiveness and decreased systemic toxicity. Notably, the combination of liposomal carfilzomib with doxorubicin (DOX) demonstrated a superior synergistic outcome than the free drug combination.^175^ Besides, combination therapy tends to perform synergistically at a specific drug ratio.^176^ The blood circulation, biodistribution, and metabolism can disturb the drug ratio when arriving at the tumor site. The application of nanomedicine‐based delivery systems ensures the maintenance of optimal synergistic ratios. Carfilzomib and DOX were loaded into liposomes at a 1:1 ratio. Although DOX and carfilzomib were released at different rates, a synergistic drug ratio was maintained between 1:1 and 2:1 in a controlled release manner and demonstrated significant tumor inhibition, achieving synergistic therapeutic efficacy.^177^

Choosing a suitable target is crucial and challenging for active targeting systems. Two target peptides, CD38 and CD138, were compared. CD138 is a heparin sulfate proteoglycan and is expressed on plasma cells. CD138 regulates the adherence and differentiation of MM cells, which enables it to be a therapeutic target. Although the binding ability of CD138‐targeted liposomes was better than that of CD38‐targeted liposomes in vitro, CD138‐targeted liposomes were prone to accumulate in the nontumor site and bind to normal cells, leading to worse performance in vivo. In addition, a long‐lasting period of in vivo study verified the superior efficacy of CD38‐targeted liposomes.^178^ A high targeting rate in vitro may not represent success in vivo, and utilization of the multivalent low‐affinity property may contribute to better binding. Chang et al. established dioleoyl phosphatidic acid (DOPA)‐based liposomes with two surface modifications, alendronate and transferrin (Tf). Both DOPA and alendronate have a bone affinity, and the Tf modification enhanced cellular uptake. This liposome demonstrated higher targeting capability and cellular uptake with pH sensitivity, around 1.6‐fold higher than the control group. Meanwhile, this system showed a higher Tf‐mediated internalization rate, enhanced apoptosis induction, and increased survival time.^179^ While there were some inconsistent results between in vitro and in vivo studies, the more complicated microenvironment in vivo may be the explanation.

Scholars added VLA‐4 antagonist and LPAM‐1 antagonist to the liposome surface to augment the selectivity of liposome. The two kinds of peptides on the surface of liposomes bound to myeloma cells cooperatively, and the optimal peptide density consisted of 0.75% VLA‐4 peptide and 1% LPAM‐1 peptide, demonstrating 28‐fold cellular uptake compared to the nontargeted liposome and approximately ten times over either one targeted liposome.^180^ Such excellent performance in vitro may promote further exploration and application of this system. BTZ was combined with Rho‐kinase inhibitor Y27632, a BM microenvironment‐disrupting agent, and both were loaded in liposomes targeting P‐selectin.^181^ Rho‐kinase is a downstream target of the MM‐BM microenvironment interaction signaling pathway, and this system attempted to target the tumor‐associated endothelium rather than tumor cells.^182^ With the precise targetability and combinational delivery in vivo, the efficacy was more dramatic than that of other formulations. This approach achieved the goal of increasing treatment sensitivity and overcoming the BM microenvironment‐induced drug resistance.

The liposomal bispecific T‐cell engager (nanoBiTE) was decorated with anti‐CD3 mAb and anti‐CD20 mAb to target T cells and tumor cells.^183^ In an aggressive Waldenstrom macroglobulinemia mouse model, the CD20/CD3 nanoBiTE group achieved eradication of the disease by day 35. Furthermore, scholars developed liposome‐based multispecific T‐cell engagers (nanoMuTEs), decorating with anti‐CD3 mAbs and multiple mAbs, including anti‐BCMA, anti‐CS1, and anti‐CD38 mAbs, against myeloma cells simultaneously. The nanoMuTEs had a long half‐life of 50–60 h, and this multispecific formulation was able to prevent the antigen‐less tumor escape. Meanwhile, nanoMuTEs exhibited a prolonged survival time in MM mouse models of approximately 10–20 days. This platform created a specific and efficacious immunotherapy strategy.

### 4.2 Micelles

Micelles are self‐assembled nanocarriers formed by amphiphilic polymers. A polymeric micelle using polyethylene glycol (PEG)‐polycaprolactone (PCL) blocks increased the stability of carfilzomib in MM cells and lung cancer cells.^184^ Zhang et al. developed core‐disulfide‐crosslinked micelles with A6 peptide‐tagged (A6‐PMs).^185^ The system increased the stability of the drug and controlled drug release according to the glutathione level. More importantly, improved tumor inhibition and better tolerance compared to the free formulation provide favorable indications for these studies. Another VLA‐4‐targeted micelle incorporated with a novel camptothecin prodrug aimed to solve cell adhesion‐mediated drug resistance. Once the nanoparticle arrived at the target, it fused with the cell membrane and transferred the payload into the cell. This system decreased tumor burden in vivo without causing severe toxicity, and provided a strategy to treat chemoresistant myeloma in combination with free chemotherapy.^186^

5‐Aza‐2ʹ‐deoxycytidine (DAC) belongs to deoxycytidine analogs, which can activate methylated and silenced genes by promoter demethylation.^187^ DAC and BTZ were encapsulated into NH_2_‐PEG‐PCL nanoparticles simultaneously. The dual‐drug micelle showed good stability and slow‐release properties, inducing reactive oxygen species (ROS) release and apoptosis at the cellular level.^188^ LP‐1 human MM cells overexpress CD44, and hyaluronic acid has a high affinity for CD44 on the cell surface. The lipophilized BTZ was encapsulated in the hyaluronic acid‐shelled and core‐disulfide‐crosslinked micelle. Because of the enhanced hydrophobic interaction, the loading efficacy was markedly increased and exhibited quick intracellular release. This complex inhibited tumor growth better than free BTZ at the same dose level, and more importantly, showed high toleration. Moreover, this platform prolonged the survival time compared with liposomal BTZ as well.^189^

### 4.3 Polymeric nanoparticles

Polymeric nanoparticles are formed by natural or synthetic polymers. The preparation process of nanoparticles can be manipulated with pH level, temperature, solvent conditions, and targetability, aiming to tailor the properties to meet variable goals. In addition, therapeutic agents can be encapsulated, conjugated, or attached to the core or surface of nanoparticles. Zhong et al. encapsulated DOX in lipoic acid‐crosslinked hyaluronic acid nanoparticles, and the disulfide crosslink structure helped stabilize the nanoparticles. The release of the DOX nanoparticle was mainly concentrated at the tumor site rather than in normal organs or tissues.^190^ Several immunotherapy modalities targeting BCMA have emerged for durable and deep responses.^191^ Nevertheless, patient‐specific procedures require complicated protocols, which limit wide applications. An off‐the‐shelf vaccine can reduce the workload and promote the activation of effective T cells. Bae et al. found an immunogenic BCMA_72‐80_ [YLMFLLRKI] peptide and developed nanoparticle‐based BCMA delivery systems. The poly(lactic‐*co*‐glycolic acid) (PLGA)‐based nanoparticles showed gradual increased uptake by antigen‐presenting cells, and exhibited the highest polyfunctional antitumor activity, with CD107a degranulation‐based cytotoxicity and production of cytokines. It evoked higher proliferation of CD8^+^cytotoxic T cells and antimyeloma effect than the free BCMA peptide.^192^ This nanoparticle‐based cancer vaccine can be utilized to maintain a long‐lasting immune response. Guo et al. formulated the PLGA/polyethyleneimine nanoparticles containing programmed cell death ligand 1 (PD‐L1) and DKK‐1 antigens.^193^ This formulation activated the responses of dendritic cells and T cells, which were essential for antitumor activity.

Active targeting strategies have excellent potential to realize precise accumulation of delivered drugs in the ideal site. Multiple avenues for active targeting strategies have been established with nanoparticles. The CD44‐specific A6 short peptide (KPSSPPEE) showed a solid affinity for CD44. Gu et al. encapsulated epirubicin into the functionalized A6‐polymersome (A6‐PS‐EPI). A6‐PS showed high drug loading ability with a simplified fabrication process. An in vivo study showed that the median survival was 240 days in the group treated with A6‐PS‐EPI and 72 days in the nontargeted PS‐EPI group, demonstrating striking efficacy.^194^ Granzyme B, the key player in NK cells, was loaded in the hyaluronic acid‐directed reduction‐responsive chimeric polymersome (HA‐RCP‐GrB). With the property of reduction‐triggered protein release and CD44 targetability, HA‐RCP‐GrB achieved a noticeable survival benefit and less weight loss than nontargeted polymersomes and blank control groups in both subcutaneous and orthotopic mouse models. Besides, decreased osteolysis in the BM was identified.^195^ In summary, these platforms improved the antitumor effect and tolerability of free drugs, providing a new avenue.

Daratumumab immunopolymersomes loaded with vincristine sulfate (Dar‐IPs‐VCR) showed superb stability, efficacious vincristine (VCR) loading rate, high targetability, and glutathione‐responsive release property. It exhibited a particular binding ability to CD38‐positive MM cells in the BM with sequential release of VCR through the glutathione‐triggered mechanism to inhibit microtubule formation and cause cell apoptosis. Dar‐IPs‐VCR completely depleted LP‐1‐Luc cells in the orthotopic MM model in vivo, with no bodyweight loss and less bone damage.^196^ Puente et al. labeled anti‐CD38 mAbs with biotin to obtain the CD38‐targeted nanoparticles loaded with BTZ. The chitosan nanoparticle had an increasable swelling property in the MM‐conditioned microenvironment, presenting with a faster release, and the 50 nm size allowed deep penetration. The CD38‐targeted nanoparticles were taken up fourfold by CD38‐positive myeloma cells than by normal cells and entered target cells through endocytosis, resulting in a much higher drug concentration.^197^ The STAT3 inhibitor is limited by its poor hydrophilic property and severe adverse events in clinical application. To overcome these defects, Huang et al. loaded S3I‐1757, a STAT3 inhibitor, into anti‐CD38 decorated nanoparticles. The researchers used a covalent thioester bond to conjugate the antibody and exhibited higher stability than biotinylation conjugations. The IC_50_ of CD38‐targeted S3I‐1757 nanoparticle was significantly lower. The nanoparticle minimized the tumor volume more efficiently than the free inhibitor, and showed increased bioavailability of the drug.^198^

Bisphosphonates are pyrophosphate analogs, and they can selectively bind to the surface of bone owing to their affinity for hydroxyapatite crystals. Bisphosphonates are also able to inhibit the progression of osteoclastic activity and are capable of inhibiting tumor cell adhesion and migration, acting as supportive agents.^199^ Due to the affinity for the bone surface of alendronate, Swami and coworkers reported a nanoparticle system with bone tissue orienting and microenvironment manipulation properties. In biodistribution studies, this bone‐targeted nanoparticle showed a 9.6‐fold accumulation compared to the nontargeted one. The targeted nanoparticle loaded with BTZ (Ald‐BTZ‐NP) contributed to bone formation and decreased tumor burden in the xenograft osteolytic bone disease model, demonstrating a promising benefit in MM treatment.^200^

### 4.4 Inorganic nanoparticles

Inorganic nanoparticles have attracted great attention in the nanomedicine area, exhibiting unique features. Metal nanoparticles have high stability, high purity, optical and electromagnetic properties, and the surface can be easily modified. Zhang et al. developed iron oxide magnetic nanoparticles (MNPs) with modified dimercaptosuccinic acid. BTZ and gambogic acid (GA) were encapsulated in MNPs. The combination platform of BTZ‐GA/MNPs showed increased inhibition of cell proliferation, as well as induction of cell apoptosis.^201^ In comparison, free BTZ and GA had a mild antitumor effect at the equivalent dose level. Since the poor water solubility limits the clinical utilization of paclitaxel, researchers synthesized paclitaxel‐loaded Fe_3_O_4_ nanoparticles (PTX‐NPs).^202^ The PTX‐NP group showed the strongest tumor volume inhibition among other groups.^203^ Moreover, superparamagnetic iron oxide nanoparticles (SPIONs) were utilized as a magnetic hyperthermia treatment, which induced endoplasmic reticulum stress and caused MM cell death.^204^ In addition, SPIONs combined tumor imaging with targeted treatment. Coating with folic acid, SPIONs selectively accumulated in the tumor site, localized the tumor by MRI, and generated heat to selectively kill tumor cells in mouse plasmacytoma models.^205^ Much work has been done to refine the properties of VCR because of its high lipophilicity and severe side‐effects.^206^ A gold nanoparticle loaded with low‐dose VCR reduced the risk of side‐effects, in which the nanomaterial acted as both a carrier and a combinational drug component, arresting cells at the S phase.^207^ The radionuclide ^89^Zr naturally homes to the bone and could be used to sensitize the therapeutic effects of drugs.^208^ Titanium dioxide (TiO_2_) nanoparticles coated with Tf and radiolabeled with ^89^Zr were able to target the BM and image the distribution of nanoparticles in mouse models. Besides, in the presence of ^89^Zr, the TiO_2_ nanoparticles were able to generate ROS and induce cell death through the apoptosis pathway.^209^

Silica is a safe and biocompatible inorganic material, and mesoporous silica nanoparticles (MSNs) are easily degraded and expelled from urine.^210^ Nigro et al. grafted folic acid on the surface of MSN and then encapsulated BTZ in the nanoparticle (FOL‐MSN‐BTZ).^211^ FOL‐MSN‐BTZ affected the metabolic pathway of MM cells by damaging mitochondrial function, decreasing ATP levels, and concurrently increasing ROS production. Meanwhile, FOL‐MSN did not impact the metabolism of normal cells, guaranteeing the safety profile of the material. This drug delivery system increased the therapeutic index and provided a safe treatment choice.

### 4.5 Other platforms

Dendrimers are a specific type of polymers with a strictly defined structure. They are globular‐shaped nanosized macromolecules with high branching, presenting with a central core and dendritic branches starting radially from the core. Besides, the outer surface of dendrimers can be modified with functional groups.^212^ BTZ is currently available only by intravenous and subcutaneous injection, with no oral formulation due to its poor oral absorption. Chaudhary et al. increased the solubility and stability of BTZ using dendrimers. They synthesized two kinds of dendrimers, G4‐PAMAM‐NH_2_ and G5‐PPI‐NH_2_, both of which can excessively improve the solubility of BTZ by more than 1000 times. And the solubility was concentration‐ and pH‐dependent through hydrophobic interactions and electrostatic interactions.^213^ Although NK cell‐based therapy has been a powerful tool to fight with malignancy, iterating infusions with a large number of NK cells after engraftment are needed. The technique of selective NK cell proliferation remains a problem.^214^ The synthetic reagents, poly(phosphorhydrazone) dendrimers, showed immunomodulatory properties in vitro, and were evidenced to activate the proliferation of NK cells. Dendrimer‐expanded NK cells were examined in subcutaneous tumor models and exhibited antitumor ability.^215^ The high reproducibility of ex vivo amplification with precise control makes it possible to be widely applied.

Hydrogels are crosslinked hydrophilic polymer chains formed networks, demonstrating a sol‐gel phase transition property.^216^ They can respond to external stimuli, including temperature, pH level, and light, according to different designs. Injectable biodegradable hydrogels have become a matter of importance for drug delivery. In addition, the incorporation of nanoparticles into hydrogels has integrated the multifunction and advantages with high tunability.^217^ To achieve sustained release of BTZ, Lee et al. encapsulated BTZ into PEG‐P(Cat)_13_ micelles with pH‐sensitive boronic ester bonds and incorporated these micelles into a hydrogel network. The BTZ‐loaded micelle/hydrogel composite served as a subcutaneously injectable and biodegradable dosage form with readily tunable mechanical properties. BTZ was released sustainedly in the acidic environment within 9 days. This composite acted as a drug reservoir and showed a meaningful delay in tumor progression after one treatment.^218^

**FIGURE 5:**
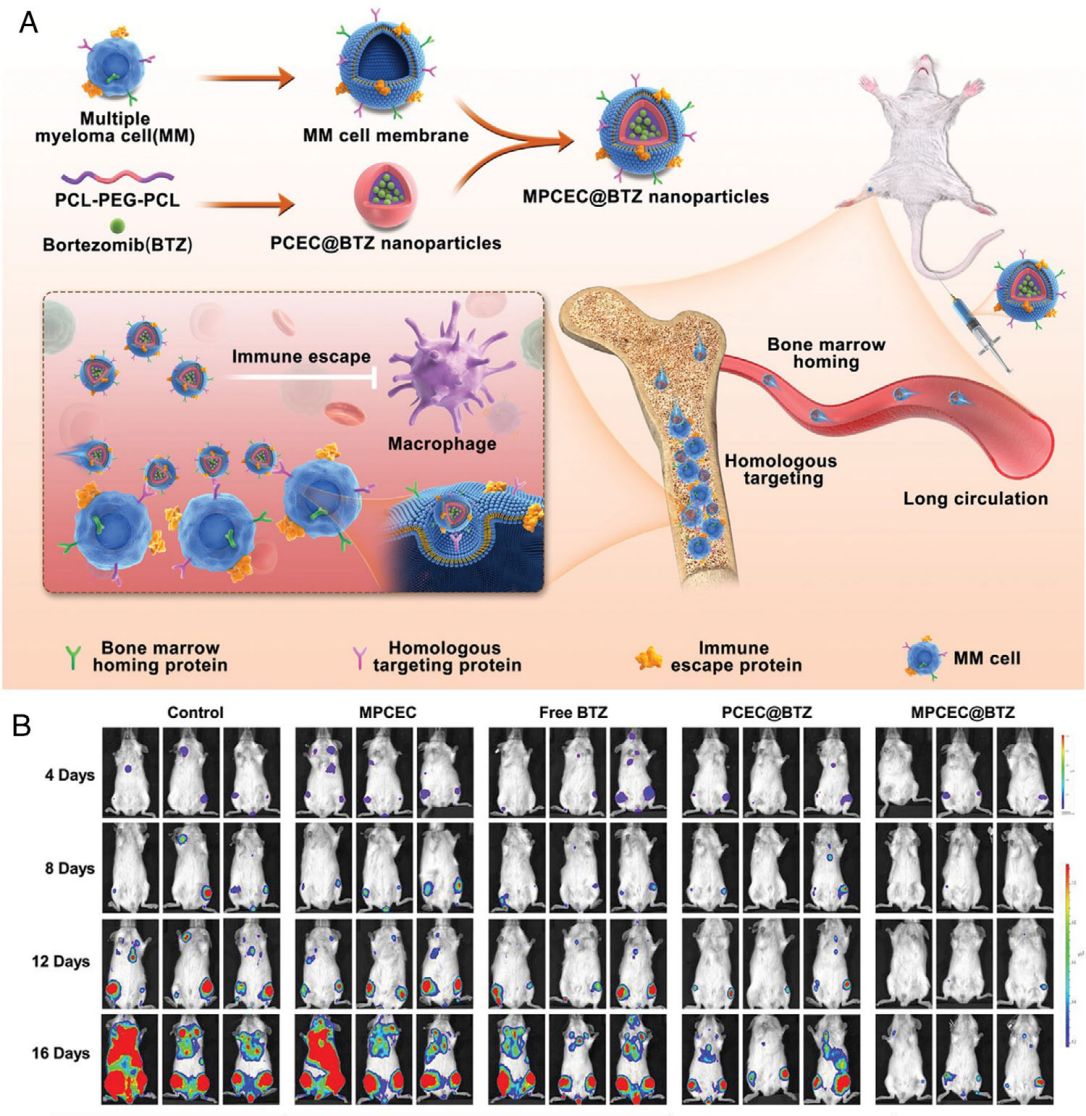
(A) Schematic illustration of MM cell membrane‐coated bortezomib (BTZ) nanoparticles for treatment of multiple myeloma. These biomimetic nanoparticles could enter the bone marrow cavity after intravenous injection, and then target tumor cells through homologous targeting. (B) Bioluminescence images of mice treated with saline (control), blank MPCEC nanoparticles, free BTZ, PCEC@BTZ nanoparticles, and MPCEC@BTZ nanoparticles (Copyright 2022, John Wiley and Sons^222^)

Biomimetic nanoparticles are novel strategies for drug delivery. Cells and their derivatives possess specific features, and nanoparticles coated with cell membranes exhibit cell‐like behaviors.^219^ Besides, these nanoparticles also retain the physicochemical properties of synthetic vehicles. Biomimetic nanoparticles are capable of immune escape, long circulation, and active targeting.^220^ Several source cells, including red blood cells, macrophages, platelets, and tumor cells, have been studied for superior targeting capacity and therapeutic efficacy. The pH‐responsive nanoparticles were coated with platelet membrane (PM‐NP), and the affinity between P‐selectin and CD44 enabled the nanoparticles to target NCI‐H929 cells. Once the nanoparticles were internalized by myeloma cells, the drugs inside were released owing to the acid‐responsive behavior. In addition, alendronate was attached to the surface of nanoparticles, aiming to accumulate in the bone site. The codelivery of BTZ and tissue plasminogen activator (tPA) enabled the PM‐NP to inhibit tumor growth and dissolve thrombus, reducing the risk of thrombus complications. The targeted‐NP‐BTZ showed higher cytotoxicity against NCI‐H929 cells with significantly lower IC_50_ value than the NP‐BTZ. Moreover, the sequential targeting strategy aimed at bone and MM cells resulted in superior treatment efficacy.^221^ MM cells mainly survive in the BM and exhibit the BM homing property. Cell membrane‐coated nanomedicine can inherit the properties of the source cell. Researchers took advantage of this phenomenon and designed an MM cell membrane‐coated BTZ nanoparticle (MPCEC@BTZ). This MM‐mimicking nanocarrier efficiently transported BTZ into BM and targeted MM cells. In addition, the system exhibited the property of immune escape. In the orthotopic MM model, the MPCEC@BTZ group demonstrated the lowest bioluminescence imaging intensity and Kappa light chain concentration. In addition, the MPCEC@BTZ formulation prolonged the median survival time of the disease model and avoided serious adverse effects (Figure 5).^222^

With the property of high spatiotemporal accuracy, photodynamic therapy can control tumor‐killing precisely.^223^, ^224^ Nevertheless, the poor penetration of external light and indeterminate tumor location limits its application in disseminated tumors. Transforming hydrophobic light‐sensitive drugs into phototherapeutic agents with tumor‐targeted lipid micelles or serum albumin nanoparticles and radiopharmaceuticals extends the application range. This strategy facilitated the treatment efficacy in the disseminated MM model with the effects of Cerenkov radiation‐induced therapy.^225^ The epirubicin‐loaded and anti‐ABCG2 mAb conjugated microbubbles, along with ultrasound exposure, attempted to eradicate the MM stem cells in the engrafted tumor mouse models. The targeted drug delivery system could anchor at the tumor site and retain for a long time; nevertheless, apart from the targeting ability, it did not enhance the therapeutic effect. When combined with ultrasound, epirubicin invaded myeloma cells facilely through membrane perforations.^226^ The preclinical results demonstrated a new strategy to target MM cells and enhance efficacy.

### 4.6 Clinical applications of nanomedicine

Nanomedicine aims to improve the therapeutic index of chemotherapeutic drugs by modifying their physicochemical properties, pharmacokinetics, and distribution in vivo. Optimized delivery of drugs to the ideal site has also been emphasized in the clinic. Several liposomes and polymeric nanoparticles have been approved to treat malignant neoplasms. The FDA has granted liposomal DOX and liposomal VCR for MM and acute lymphoblastic leukemia. Liposomal DOX achieved long circulation and preferential accumulation at the tumor site, attenuating cardiac toxicity in both animal models and humans.^227^ The PLD has been evaluated in different combination regimens including PIs or IMiDs. The BTZ, PLD, and dexamethasone regimen seems highly effective for initial therapy. In a pivotal trial, patients who received PLD and BTZ showed superior effects than the BTZ monotherapy group in the interim analysis.^228^ However, the final results indicated that this combination did not improve OS compared to monotherapy in long‐term follow‐up.^229^ CPX‐351 gains satisfying outcomes as a dual‐drug liposomal formulation that encapsulates daunorubicin and cytarabine at a fixed ratio of 5:1.^230^ The results of clinical trials demonstrated clinical benefits and confirmed CPX‐351 as a standard intensive therapy for high‐risk or secondary acute myeloid leukemia in older patients.^231^ Although nanomedicine has shown inspiring efficacy in preclinical studies, many limitations and difficulties remain in the path of clinical translation. The limited gain in OS has not satisfied clinicians and patients. Rare active targeting nanoparticles have been applied in the clinic so far. The complicated preparation process restricts large‐scale output. In addition, clinical trials and long‐term outcomes are required to verify the efficacy and safety of nanomedicine.

### 4.7 Challenges of nanomedicine in multiple myeloma

Nanomedicine has demonstrated promising efficacy in tumor treatment. Several factors are essential for designing nanomedicine including preparation methods, different targeting peptides or antibodies, length, and hydrophilicity of conjugated linkers.^232^ In addition, excellent performance in vitro cannot guarantee a satisfying outcome in vivo. The complicated situation in vivo, including blood circulation, biodistribution, tumor cell penetration, and the interaction between nanoparticles and healthy tissues, hindered the voyage to the target site, leading to disappointing behavior.^233^ In vivo estimation remains crucial for clinical application. The hydrophilicity of the targeting peptides, the valency of the peptides on the surface, and nanoparticle size matter greatly. Therefore, upgrading these parameters and finding the balance among these factors is essential to uplift the in vivo therapeutic results.

MM is highly heterogeneous, and stemming from the heterogeneous expression levels of surface molecules on MM cells, there still exist limitations to achieving precise targeting. Therefore, digging the highly unique surface marker of MM, creating dual‐targeted nanoparticles, and optimizing the preparation are essential steps to improve the efficacy. Other significant issues of MM are drug resistance and disease relapse. Many patients suffer from relapse, showing double or even triple refractory. Mapping out a strategy to achieve MRD‐negativity may be the key point to solve the problem. Several organic nanoparticles formulated by polymeric materials have been widely studied and have shown high biocompatibility, biodegradability, and low toxicity based on tests in cells and animal models. Because of the different characteristics of ingredients and surface modifications, the metabolic pathways of nanomedicine in vivo vary from each other. Therefore, safety issues should be considered. The long‐term effects of nanomaterials are essential for biosafety assessment and clinical application but are currently less explored.

The two main myeloma animal models are xenogeneic model and syngeneic tumor model. Culturing myeloma cells in human fetal bone or rabbit bone and then implanting them into severe combined immune deficiency mice can sustain myeloma cell growth and recreate the look‐alike microenvironment.^234^ Syngeneic tumor models, such as the transplantable murine model 5T33 and genetically engineered mouse models, can present aggressive late‐stage disease.^235^ Gu et al. established an orthotopic MM mouse model by intravenous injection of LP‐1‐Luc MM cells, which were disseminated to the whole body.^194^ Another A6 peptide targeted nanoparticle was studied in a subcutaneous model with an implanted human BM‐like scaffold, providing an advanced manner to verify the effectiveness of micelle‐drug formulations.^185^ However, subcutaneous xenograft mouse models are mostly used for their viability and convenience, although they cannot genuinely imitate the complicated microenvironment. Even though nanoparticles showed validation in these models, they may not demonstrate matching efficacy in the clinical situation. Establishing orthotopic animal models and patient‐derived xenograft models can be more reliable to verify the power of nanomedicine.

## 5 CONCLUSIONS AND PERSPECTIVES

Improved understanding of biological development, molecular abnormalities, and the BM microenvironment of MM enables effective treatment and management for patients. To date, the survival of MM patients has improved as large amount of therapeutic agents have emerged. Next‐generation PIs, IMiDs, mAbs, CAR T‐cell therapies, and novel agents are favorable advancements, and ASCT is still a cornerstone during management. In addition to currently adopted approaches, drug delivery strategies should be considered. Nanomedicine can meliorate the pharmacokinetic and pharmacodynamic properties of conventional agents. Nanoplatforms loaded with cytotoxic drugs and PIs have achieved controlled drug release, prolonged circulation, and reduced systemic toxicity. Active targeting nanoparticles with particular affinity for MM cells, bone, or BM have been designed for precise treatment (Table 3). Besides, nanomedicine acts as an aide in immunotherapy to strengthen immune effects. However, challenges still exist, and a portion of patients have poor outcomes.

**TABLE 3:** Studies of active targeting nanoparticles for multiple myeloma

Targets	Types	Agents	Injection method	In vivo model	Refs.
CD38	Liposome	Doxorubicin	iv	NCI‐H929 subcutaneous model	178
Bone/transferrin receptor	Liposome	Paclitaxel	ip	MM.1S orthotopic model	179
P‐selectin	Liposome	Bortezomib/Y27632	iv	MM.1S orthotopic model	181
CD3/BCMA, CS1, CD38	Liposomal multispecific T‐cell engager	–	iv	MM.1S orthotopic model	183
CD44	Core‐disulfide‐crosslinked micelle	Carfilzomib	iv	LP‐1 subcutaneous model	185
VLA‐4	Micelle	Camptothecin prodrug	iv	5TGM1 orthotopic model	186
CD44	Hyaluronic acid‐shelled and core‐disulfide‐crosslinked micelle	Bortezomib	iv	LP‐1 subcutaneous model	192
CD44	Lipoic acid‐crosslinked hyaluronic acid nanoparticle	Doxorubicin	iv	LP‐1 subcutaneous model	190
CD44	Polymersome	Epirubicin	iv	LP‐1 orthotopic model	194
CD44	Chimeric polymersome	Granzyme B	iv	LP‐1 subcutaneous/orthotopic model	195
CD38	Immunopolymersome	Vincristine sulfate	iv	LP‐1 orthotopic model	196
CD38	Chitosan nanoparticle	Bortezomib	iv	MM.1S orthotopic model	197
CD38	Polymeric nanoparticle	S3I‐1757	iv	U266 orthotopic model	198
Bone	Polymeric nanoparticle	Bortezomib	ip	MM.1S orthotopic model	200
Bone marrow/transferrin receptor	Titanium dioxide nanoparticle	^89^Zr	iv	MM.1S orthotopic model	209
Bone/CD44	Platelet membrane‐coated nanoparticle	Bortezomib/tPA	iv	NCI‐H929 orthotopic model	221
Bone marrow	MM cell membrane‐coated nanoparticle	Bortezomib	iv	MRD orthotopic model	222

Abbreviations. ip, intraperitoneal injection; iv, intravenous injection.

When designing treatment strategies, personalized situations, tolerability, and molecular information should be taken into account. Patients with high‐risk features need to be identified early, and the application of biomarkers can help to select subgroups for appropriate treatment. Besides, real‐world evidence is needed to assess the benefits, toxicity, and long‐term effects. Studies are underway to figure out potential combination strategies, mechanisms of drug resistance, new targets, and new drug classes. Regarding the further development of nanomedicine, several aspects deserve the attention of researchers. The precision, efficacy, and safety of nanomedicine systems are crucial issues, thus, requiring rational and ingenious design. Combination therapies, multiple targeting strategies, and immunotherapies are promising paths for nanotechnology application. In addition, orthotopic animal models are needed as standards to judge the effect of nanomedicine platforms. Moreover, the convenience of large‐scale manufacture and long‐term storage for clinical translation should be considered as well.

## CONFLICT OF INTEREST

Zhiyong Qian is an editorial board member of MedComm. Author Zhiyong Qian was not involved in the journal's review of, or decisions related to, this manuscript. The other authors declared no conflict of interest.

## AUTHOR CONTRIBUTIONS

Ting Niu and Zhiyong Qian conceived the ideas of the manuscript and supervised the writing. Peipei Yang and Ying Qu drafted and revised the manuscript. Mengyao Wang, Bingyang Chu, Yuhuan Zheng, and Wen Chen edited the manuscript and summarized related research. All authors read and approved the final manuscript.

## ETHICS APPROVAL

Not applicable.

## Data Availability

Not applicable.

## References

[1] Palumbo A , Anderson K . Multiple myeloma. N Engl J Med. 2011;364(11):1046‐1060. 21410373 10.1056/NEJMra1011442

[2] Siegel RL , Miller KD , Fuchs HE , Jemal A . Cancer statistics, 2021. CA Cancer J Clin. 2021;71(1):7‐33. 33433946 10.3322/caac.21654

[3] Zhou L , Yu Q , Wei G , et al. Measuring the global, regional, and national burden of multiple myeloma from 1990 to 2019. BMC Cancer. 2021;21(1):606. 34034700 10.1186/s12885-021-08280-y PMC8152089

[4] van de Donk N , Pawlyn C , Yong KL . Multiple myeloma. Lancet. 2021;397(10272):410‐427. 33516340 10.1016/S0140-6736(21)00135-5

[5] Musto P , Engelhardt M , Caers J , et al. European Myeloma Network review and consensus statement on smoldering multiple myeloma: how to distinguish (and manage) Dr. Jekyll and Mr. Hyde. Haematologica. 2021;106(11):2799‐2812. 34261295 10.3324/haematol.2021.278519 PMC8561280

[6] Ravindran A , Bartley AC , Holton SJ , et al. Prevalence, incidence and survival of smoldering multiple myeloma in the United States. Blood Cancer J. 2016;6(10):e486. 27768092 10.1038/bcj.2016.100 PMC5098258

[7] Kyle RA , Remstein ED , Therneau TM , et al. Clinical course and prognosis of smoldering (asymptomatic) multiple myeloma. N Engl J Med. 2007;356(25):2582‐2590. 17582068 10.1056/NEJMoa070389

[8] Raje NS , Bhatta S , Terpos E . Role of the RANK/RANKL pathway in multiple myeloma. Clin Cancer Res. 2019;25(1):12‐20. 30093448 10.1158/1078-0432.CCR-18-1537

[9] Bazarbachi AH , Al Hamed R , Malard F , Harousseau JL , Mohty M . Relapsed refractory multiple myeloma: a comprehensive overview. Leukemia. 2019;33(10):2343‐2357. 31455853 10.1038/s41375-019-0561-2

[10] Chakraborty R , Majhail NS . Treatment and disease‐related complications in multiple myeloma: implications for survivorship. Am J Hematol. 2020;95(6):672‐690. 32086970 10.1002/ajh.25764 PMC7217756

[11] Kumar SK , Rajkumar V , Kyle RA , et al. Multiple myeloma. Nat Rev Dis Primers. 2017;3:17046. 28726797 10.1038/nrdp.2017.46

[12] Bianchi G , Munshi NC . Pathogenesis beyond the cancer clone(s) in multiple myeloma. Blood. 2015;125(20):3049‐3058. 25838343 10.1182/blood-2014-11-568881 PMC4432002

[13] Bergsagel PL , Kuehl WM , Zhan F , Sawyer J , Barlogie B , Shaughnessy J Jr . Cyclin D dysregulation: an early and unifying pathogenic event in multiple myeloma. Blood. 2005;106(1):296‐303. 15755896 10.1182/blood-2005-01-0034 PMC1895118

[14] Morgan GJ , Walker BA , Davies FE . The genetic architecture of multiple myeloma. Nat Rev Cancer. 2012;12(5):335‐348. 22495321 10.1038/nrc3257

[15] Avet‐Loiseau H , Attal M , Moreau P , et al. Genetic abnormalities and survival in multiple myeloma: the experience of the Intergroupe Francophone du Myélome. Blood. 2007;109(8):3489‐3495. 17209057 10.1182/blood-2006-08-040410

[16] Rajkumar SV , Gupta V , Fonseca R , et al. Impact of primary molecular cytogenetic abnormalities and risk of progression in smoldering multiple myeloma. Leukemia. 2013;27(8):1738‐1744. 23515097 10.1038/leu.2013.86 PMC3773463

[17] Neuse CJ , Lomas OC , Schliemann C , et al. Genome instability in multiple myeloma. Leukemia. 2020;34(11):2887‐2897. 32651540 10.1038/s41375-020-0921-y

[18] Shaughnessy J Jr. , Gabrea A , Qi Y , et al. Cyclin D3 at 6p21 is dysregulated by recurrent chromosomal translocations to immunoglobulin loci in multiple myeloma. Blood. 2001;98(1):217‐223. 11418483 10.1182/blood.v98.1.217

[19] Avet‐Loiseau H , Malard F , Campion L , et al. Translocation t(14;16) and multiple myeloma: is it really an independent prognostic factor? Blood. 2011;117(6):2009‐2011. 20962323 10.1182/blood-2010-07-295105

[20] Goldman‐Mazur S , Jurczyszyn A , Castillo JJ , et al. A multicenter retrospective study of 223 patients with t(14;16) in multiple myeloma. Am J Hematol. 2020;95(5):503‐509. 32072687 10.1002/ajh.25758

[21] Vekemans MC , Lemmens H , Delforge M , et al. The t(14;20)(q32;q12): a rare cytogenetic change in multiple myeloma associated with poor outcome. Br J Haematol. 2010;149(6):901‐904. 20148877 10.1111/j.1365-2141.2010.08113.x

[22] Chretien ML , Corre J , Lauwers‐Cances V , et al. Understanding the role of hyperdiploidy in myeloma prognosis: which trisomies really matter? Blood. 2015;126(25):2713‐2719. 26516228 10.1182/blood-2015-06-650242 PMC4683332

[23] Lakshman A , Painuly U , Rajkumar SV , et al. Impact of acquired del(17p) in multiple myeloma. Blood Adv. 2019;3(13):1930‐1938. 31248884 10.1182/bloodadvances.2018028530 PMC6616261

[24] Corre J , Perrot A , Caillot D , et al. del(17p) without TP53 mutation confers a poor prognosis in intensively treated newly diagnosed patients with multiple myeloma. Blood. 2021;137(9):1192‐1195. 33080624 10.1182/blood.2020008346 PMC7933766

[25] Schmidt TM , Fonseca R , Usmani SZ . Chromosome 1q21 abnormalities in multiple myeloma. Blood Cancer J. 2021;11(4):83. 33927196 10.1038/s41408-021-00474-8 PMC8085148

[26] Burroughs Garcìa J , Eufemiese RA , Storti P , et al. Role of 1q21 in multiple myeloma: from pathogenesis to possible therapeutic targets. Cells. 2021;10(6):1360. 34205916 10.3390/cells10061360 PMC8227721

[27] Walker BA , Boyle EM , Wardell CP , et al. Mutational spectrum, copy number changes, and outcome: results of a sequencing study of patients with newly diagnosed myeloma. J Clin Oncol. 2015;33(33):3911‐3920. 26282654 10.1200/JCO.2014.59.1503 PMC6485456

[28] Walker BA , Mavrommatis K , Wardell CP , et al. Identification of novel mutational drivers reveals oncogene dependencies in multiple myeloma. Blood. 2018;132(6):587‐597. 29884741 10.1182/blood-2018-03-840132 PMC6097138

[29] Sharma N , Smadbeck JB , Abdallah N , et al. The prognostic role of MYC structural variants identified by NGS and FISH in multiple myeloma. Clin Cancer Res. 2021;27(19):5430‐5439. 34233962 10.1158/1078-0432.CCR-21-0005 PMC8738776

[30] Morgan GJ , He J , Tytarenko R , et al. Kinase domain activation through gene rearrangement in multiple myeloma. Leukemia. 2018;32(11):2435‐2444. 29654269 10.1038/s41375-018-0108-y PMC6224403

[31] Roy P , Sarkar UA , Basak S . The NF‐κB activating pathways in multiple myeloma. Biomedicines. 2018;6(2):59. 10.3390/biomedicines6020059 PMC6027071 29772694

[32] Vrábel D , Pour L , Ševčíková S . The impact of NF‐κB signaling on pathogenesis and current treatment strategies in multiple myeloma. Blood Rev. 2019;34:56‐66. 30501907 10.1016/j.blre.2018.11.003

[33] Boyle EM , Deshpande S , Tytarenko R , et al. The molecular make up of smoldering myeloma highlights the evolutionary pathways leading to multiple myeloma. Nat Commun. 2021;12(1):293. 33436579 10.1038/s41467-020-20524-2 PMC7804406

[34] Drosten M , Barbacid M . Targeting the MAPK pathway in KRAS‐driven tumors. Cancer Cell. 2020;37(4):543‐550. 32289276 10.1016/j.ccell.2020.03.013

[35] Shirazi F , Jones RJ , Singh RK , et al. Activating KRAS, NRAS, and BRAF mutants enhance proteasome capacity and reduce endoplasmic reticulum stress in multiple myeloma. Proc Natl Acad Sci U S A. 2020;117(33):20004‐20014. 32747568 10.1073/pnas.2005052117 PMC7443929

[36] Kharaziha P , De Raeve H , Fristedt C , et al. Sorafenib has potent antitumor activity against multiple myeloma in vitro, ex vivo, and in vivo in the 5T33MM mouse model. Cancer Res. 2012;72(20):5348‐5362. 22952216 10.1158/0008-5472.CAN-12-0658

[37] Barwick BG , Gupta VA , Vertino PM , Boise LH . Cell of origin and genetic alterations in the pathogenesis of multiple myeloma. Front Immunol. 2019;10:1121. 31231360 10.3389/fimmu.2019.01121 PMC6558388

[38] Martínez‐Moreno M , Leiva M , Aguilera‐Montilla N , et al. In vivo adhesion of malignant B cells to bone marrow microvasculature is regulated by α4β1 cytoplasmic‐binding proteins. Leukemia. 2016;30(4):861‐872. 26658839 10.1038/leu.2015.332

[39] García‐Ortiz A , Rodríguez‐García Y , Encinas J , et al. The role of tumor microenvironment in multiple myeloma development and progression. Cancers (Basel). 2021;13(2):217. 10.3390/cancers13020217 PMC7827690 33435306

[40] Lindquist RL , Niesner RA , HauserAE . In the right place, at the right time: spatiotemporal conditions determining plasma cell survival and function. Front Immunol. 2019;10:788. 31068930 10.3389/fimmu.2019.00788 PMC6491733

[41] Akhtar S , Ali TA , Faiyaz A , et al. Cytokine‐mediated dysregulation of signaling pathways in the pathogenesis of multiple myeloma. Int J Mol Sci. 2020;21(14):5002. 10.3390/ijms21145002 PMC7403981 32679860

[42] Sprynski AC , Hose D , Caillot L , et al. The role of IGF‐1 as a major growth factor for myeloma cell lines and the prognostic relevance of the expression of its receptor. Blood. 2009;113(19):4614‐4626. 19228610 10.1182/blood-2008-07-170464 PMC2691749

[43] Kolosenko I , Grander D , Tamm KP . IL‐6 activated JAK/STAT3 pathway and sensitivity to Hsp90 inhibitors in multiple myeloma. Curr Med Chem. 2014;21(26):3042‐3047. 24735367 10.2174/0929867321666140414100831

[44] Hideshima T , Anderson KC . Signaling pathway mediating myeloma cell growth and survival. Cancers (Basel). 2021;13(2):216. 10.3390/cancers13020216 PMC7827005 33435632

[45] Ria R , Vacca A . Bone marrow stromal cells‐induced drug resistance in multiple myeloma. Int J Mol Sci. 2020;21(2):613. 10.3390/ijms21020613 PMC7013615 31963513

[46] Görgün GT , Whitehill G , Anderson JL , et al. Tumor‐promoting immune‐suppressive myeloid‐derived suppressor cells in the multiple myeloma microenvironment in humans. Blood. 2013;121(15):2975‐2987. 23321256 10.1182/blood-2012-08-448548 PMC3624943

[47] Holthof LC , Mutis T . Challenges for immunotherapy in multiple myeloma: bone marrow microenvironment‐mediated immune suppression and immune resistance. Cancers (Basel). 2020;12(4):988. 10.3390/cancers12040988 PMC7226482 32316450

[48] Li M , Xia B , Wang Y , You MJ , Zhang Y . Potential therapeutic roles of exosomes in multiple myeloma: a systematic review. J Cancer. 2019;10(24):6154‐6160. 31762825 10.7150/jca.31752 PMC6856585

[49] Roccaro AM , Sacco A , Maiso P , et al. BM mesenchymal stromal cell‐derived exosomes facilitate multiple myeloma progression. J Clin Invest. 2013;123(4):1542‐1555. 23454749 10.1172/JCI66517 PMC3613927

[50] Raimondo S , Saieva L , Vicario E , et al. Multiple myeloma‐derived exosomes are enriched of amphiregulin (AREG) and activate the epidermal growth factor pathway in the bone microenvironment leading to osteoclastogenesis. J Hematol Oncol. 2019;12(1):2. 30621731 10.1186/s13045-018-0689-y PMC6325886

[51] Wang J , De Veirman K , Faict S , et al. Multiple myeloma exosomes establish a favourable bone marrow microenvironment with enhanced angiogenesis and immunosuppression. J Pathol. 2016;239(2):162‐173. 26956697 10.1002/path.4712

[52] Xu H , Han H , Song S , et al. Exosome‐transmitted PSMA3 and PSMA3‐AS1 promote proteasome inhibitor resistance in multiple myeloma. Clin Cancer Res. 2019;25(6):1923‐1935. 30610101 10.1158/1078-0432.CCR-18-2363

[53] Terpos E , Ntanasis‐Stathopoulos I , Gavriatopoulou M , Dimopoulos MA . Pathogenesis of bone disease in multiple myeloma: from bench to bedside. Blood Cancer J. 2018;8(1):7. 29330358 10.1038/s41408-017-0037-4 PMC5802524

[54] Goranova‐Marinova V , Goranov S , Pavlov P , Tzvetkova T . Serum levels of OPG, RANKL and RANKL/OPG ratio in newly‐diagnosed patients with multiple myeloma. Clinical correlations. Haematologica. 2007;92(7):1000‐1001. 17606458 10.3324/haematol.10943

[55] Tsirakis G , Roussou P , Pappa CA , et al. Increased serum levels of MIP‐1alpha correlate with bone disease and angiogenic cytokines in patients with multiple myeloma. Med Oncol. 2014;31(1):778. 24277416 10.1007/s12032-013-0778-2

[56] Mukkamalla SKR , Malipeddi D . Myeloma bone disease: a comprehensive review. Int J Mol Sci. 2021;22(12):6208. 34201396 10.3390/ijms22126208 PMC8227693

[57] van Andel H , Kocemba KA , Spaargaren M , Pals ST . Aberrant Wnt signaling in multiple myeloma: molecular mechanisms and targeting options. Leukemia. 2019;33(5):1063‐1075. 30770859 10.1038/s41375-019-0404-1 PMC6756057

[58] Zhou F , Meng S , Song H , Claret FX . Dickkopf‐1 is a key regulator of myeloma bone disease: opportunities and challenges for therapeutic intervention. Blood Rev. 2013;27(6):261‐267. 24054128 10.1016/j.blre.2013.08.002 PMC4133945

[59] Panaroni C , Yee AJ , Raje NS . Myeloma and bone disease. Curr Osteoporos Rep. 2017;15(5):483‐498. 28861842 10.1007/s11914-017-0397-5

[60] Rosiñol L , Oriol A , Rios R , et al. Bortezomib, lenalidomide, and dexamethasone as induction therapy prior to autologous transplant in multiple myeloma. Blood. 2019;134(16):1337‐1345. 31484647 10.1182/blood.2019000241 PMC6888142

[61] Rajkumar SV . Multiple myeloma: 2020 update on diagnosis, risk‐stratification and management. Am J Hematol. 2020;95(5):548‐567. 32212178 10.1002/ajh.25791

[62] Durer C , Durer S , Lee S , et al. Treatment of relapsed multiple myeloma: evidence‐based recommendations. Blood Rev. 2020;39:100616. 31500848 10.1016/j.blre.2019.100616

[63] Attal M , Richardson PG , Rajkumar SV , et al. Isatuximab plus pomalidomide and low‐dose dexamethasone versus pomalidomide and low‐dose dexamethasone in patients with relapsed and refractory multiple myeloma (ICARIA‐MM): a randomised, multicentre, open‐label, phase 3 study. Lancet. 2019;394(10214):2096‐2107. 31735560 10.1016/S0140-6736(19)32556-5

[64] Manasanch EE , Orlowski RZ . Proteasome inhibitors in cancer therapy. Nat Rev Clin Oncol. 2017;14(7):417‐433. 28117417 10.1038/nrclinonc.2016.206 PMC5828026

[65] Albornoz N , Bustamante H , Soza A , Burgos P . Cellular responses to proteasome inhibition: molecular mechanisms and beyond. Int J Mol Sci. 2019;20(14):3379. 10.3390/ijms20143379 PMC6678303 31295808

[66] Narayanan S , Cai CY , Assaraf YG , et al. Targeting the ubiquitin‐proteasome pathway to overcome anti‐cancer drug resistance. Drug Resist Updat. 2020;48:100663. 31785545 10.1016/j.drup.2019.100663

[67] Ito S . Proteasome inhibitors for the treatment of multiple myeloma. Cancers (Basel). 2020;12(2):265. 10.3390/cancers12020265 PMC7072336 31979059

[68] Gay F , Magarotto V , Crippa C , et al. Bortezomib induction, reduced‐intensity transplantation, and lenalidomide consolidation‐maintenance for myeloma: updated results. Blood. 2013;122(8):1376‐1383. 23775712 10.1182/blood-2013-02-483073

[69] Durie BGM , Hoering A , Abidi MH , et al. Bortezomib with lenalidomide and dexamethasone versus lenalidomide and dexamethasone alone in patients with newly diagnosed myeloma without intent for immediate autologous stem‐cell transplant (SWOG S0777): a randomised, open‐label, phase 3 trial. Lancet. 2017;389(10068):519‐527. 28017406 10.1016/S0140-6736(16)31594-X PMC5546834

[70] Durie BGM , Hoering A , Sexton R , et al. Longer term follow‐up of the randomized phase III trial SWOG S0777: bortezomib, lenalidomide and dexamethasone vs. lenalidomide and dexamethasone in patients (Pts) with previously untreated multiple myeloma without an intent for immediate autologous stem cell transplant (ASCT). Blood Cancer J. 2020;10(5):53. 32393732 10.1038/s41408-020-0311-8 PMC7214419

[71] Salwender H , Elmaagacli A , Merz M , et al. Long‐term follow‐up of subcutaneous versus intravenous bortezomib during induction therapy for newly diagnosed multiple myeloma treated within the GMMG‐MM5 Phase III Trial. Leukemia. 2021;35(10):3007‐3011. 34031532 10.1038/s41375-021-01298-y

[72] Kortuem KM , Stewart AK . Carfilzomib. Blood. 2013;121(6):893‐897. 23393020 10.1182/blood-2012-10-459883

[73] Facon T , Niesvizky R , Mateos MV , et al. Efficacy and safety of carfilzomib‐based regimens in frail patients with relapsed and/or refractory multiple myeloma. Blood Adv. 2020;4(21):5449‐5459. 33166401 10.1182/bloodadvances.2020001965 PMC7656926

[74] Siegel DS , Martin T , Wang M , et al. A phase 2 study of single‐agent carfilzomib (PX‐171‐003‐A1) in patients with relapsed and refractory multiple myeloma. Blood. 2012;120(14):2817‐2825. 22833546 10.1182/blood-2012-05-425934 PMC4123387

[75] Stewart AK , Rajkumar SV , Dimopoulos MA , et al. Carfilzomib, lenalidomide, and dexamethasone for relapsed multiple myeloma. N Engl J Med. 2015;372(2):142‐152. 25482145 10.1056/NEJMoa1411321

[76] Kumar SK , Jacobus SJ , Cohen AD , et al. Carfilzomib or bortezomib in combination with lenalidomide and dexamethasone for patients with newly diagnosed multiple myeloma without intention for immediate autologous stem‐cell transplantation (ENDURANCE): a multicentre, open‐label, phase 3, randomised, controlled trial. Lancet Oncol. 2020;21(10):1317‐1330. 32866432 10.1016/S1470-2045(20)30452-6 PMC7591827

[77] Siegel D , Martin T , Nooka A , et al. Integrated safety profile of single‐agent carfilzomib: experience from 526 patients enrolled in 4 phase II clinical studies. Haematologica. 2013;98(11):1753‐1761. 23935022 10.3324/haematol.2013.089334 PMC3815177

[78] Shirley M . Ixazomib: first global approval. Drugs. 2016;76(3):405‐411. 26846321 10.1007/s40265-016-0548-5

[79] Moreau P , Masszi T , Grzasko N , et al. Oral ixazomib, lenalidomide, and dexamethasone for multiple myeloma. N Engl J Med. 2016;374(17):1621‐1634. 27119237 10.1056/NEJMoa1516282

[80] Avet‐Loiseau H , Bahlis NJ , Chng WJ , et al. Ixazomib significantly prolongs progression‐free survival in high‐risk relapsed/refractory myeloma patients. Blood. 2017;130(24):2610‐2618. 29054911 10.1182/blood-2017-06-791228

[81] Facon T , Venner CP , Bahlis NJ , et al. Oral ixazomib, lenalidomide, and dexamethasone for transplant‐ineligible patients with newly diagnosed multiple myeloma. Blood. 2021;137(26):3616‐3628. 33763699 10.1182/blood.2020008787 PMC8462404

[82] Richardson PG , Zimmerman TM , Hofmeister CC , et al. Phase 1 study of marizomib in relapsed or relapsed and refractory multiple myeloma: nPI‐0052‐101 Part 1. Blood. 2016;127(22):2693‐2700. 27009059 10.1182/blood-2015-12-686378 PMC5413296

[83] Hari P , Matous JV , Voorhees PM , et al. Oprozomib in patients with newly diagnosed multiple myeloma. Blood Cancer J. 2019;9(9):66. 31420532 10.1038/s41408-019-0232-6 PMC6697695

[84] Lu G , Middleton RE , Sun H , et al. The myeloma drug lenalidomide promotes the cereblon‐dependent destruction of Ikaros proteins. Science. 2014;343(6168):305‐309. 24292623 10.1126/science.1244917 PMC4070318

[85] Asatsuma‐Okumura T , Ito T , Handa H . Molecular mechanisms of cereblon‐based drugs. Pharmacol Ther. 2019;202:132‐139. 31202702 10.1016/j.pharmthera.2019.06.004

[86] Quach H , Ritchie D , Stewart AK , et al. Mechanism of action of immunomodulatory drugs (IMiDS) in multiple myeloma. Leukemia. 2010;24(1):22‐32. 19907437 10.1038/leu.2009.236 PMC3922408

[87] Tacchetti P , Pantani L , Patriarca F , et al. Bortezomib, thalidomide, and dexamethasone followed by double autologous haematopoietic stem‐cell transplantation for newly diagnosed multiple myeloma (GIMEMA‐MMY‐3006): long‐term follow‐up analysis of a randomised phase 3, open‐label study. Lancet Haematol. 2020;7(12):e861‐e873. 33242443 10.1016/S2352-3026(20)30323-9

[88] Galustian C , Meyer B , Labarthe MC , et al. The anti‐cancer agents lenalidomide and pomalidomide inhibit the proliferation and function of T regulatory cells. Cancer Immunol Immunother. 2009;58(7):1033‐1045. 19009291 10.1007/s00262-008-0620-4 PMC11030759

[89] Dimopoulos M , Spencer A , Attal M , et al. Lenalidomide plus dexamethasone for relapsed or refractory multiple myeloma. N Engl J Med. 2007;357(21):2123‐2132. 18032762 10.1056/NEJMoa070594

[90] Korde N , Roschewski M , Zingone A , et al. Treatment with carfilzomib‐lenalidomide‐dexamethasone with lenalidomide extension in patients with smoldering or newly diagnosed multiple myeloma. JAMA Oncol. 2015;1(6):746‐754. 26181891 10.1001/jamaoncol.2015.2010 PMC6662597

[91] Voorhees PM , Kaufman JL , Laubach J , et al. Daratumumab, lenalidomide, bortezomib, and dexamethasone for transplant‐eligible newly diagnosed multiple myeloma: the GRIFFIN trial. Blood. 2020;136(8):936‐945. 32325490 10.1182/blood.2020005288 PMC7441167

[92] Elkinson S , McCormack PL . Pomalidomide: first global approval. Drugs. 2013;73(6):595‐604. 23572409 10.1007/s40265-013-0047-x

[93] Richardson PG , Siegel DS , Vij R , et al. Pomalidomide alone or in combination with low‐dose dexamethasone in relapsed and refractory multiple myeloma: a randomized phase 2 study. Blood. 2014;123(12):1826‐1832. 24421329 10.1182/blood-2013-11-538835 PMC3962162

[94] Baz RC , Martin TG , 3rd, Lin HY , et al. Randomized multicenter phase 2 study of pomalidomide, cyclophosphamide, and dexamethasone in relapsed refractory myeloma. Blood. 2016;127(21):2561‐2568. 26932802 10.1182/blood-2015-11-682518

[95] Paludo J , Mikhael JR , LaPlant BR , et al. Pomalidomide, bortezomib, and dexamethasone for patients with relapsed lenalidomide‐refractory multiple myeloma. Blood. 2017;130(10):1198‐1204. 28684537 10.1182/blood-2017-05-782961 PMC5606008

[96] Richardson PG , Oriol A , Beksac M , et al. Pomalidomide, bortezomib, and dexamethasone for patients with relapsed or refractory multiple myeloma previously treated with lenalidomide (OPTIMISMM): a randomised, open‐label, phase 3 trial. Lancet Oncol. 2019;20(6):781‐794. 31097405 10.1016/S1470-2045(19)30152-4

[97] Shah JJ , Stadtmauer EA , Abonour R , et al. Carfilzomib, pomalidomide, and dexamethasone for relapsed or refractory myeloma. Blood. 2015;126(20):2284‐2290. 26384354 10.1182/blood-2015-05-643320 PMC4643003

[98] Bringhen S , Mina R , Cafro AM , et al. Once‐weekly carfilzomib, pomalidomide, and low‐dose dexamethasone for relapsed/refractory myeloma: a phase I/II study. Leukemia. 2018;32(8):1803‐1807. 29479061 10.1038/s41375-018-0024-1

[99] Morandi F , Airoldi I , Marimpietri D , Bracci C , Faini AC , Gramignoli R . CD38, a receptor with multifunctional activities: from modulatory functions on regulatory cell subsets and extracellular vesicles, to a target for therapeutic strategies. Cells. 2019;8(12):1527. 10.3390/cells8121527 PMC6953043 31783629

[100] Bonello F , D'Agostino M , Moscvin M , Cerrato C , Boccadoro M , Gay F . CD38 as an immunotherapeutic target in multiple myeloma. Expert Opin Biol Ther. 2018;18(12):1209‐1221. 30394809 10.1080/14712598.2018.1544240

[101] Sanchez L , Wang Y , Siegel DS , Wang ML . Daratumumab: a first‐in‐class CD38 monoclonal antibody for the treatment of multiple myeloma. J Hematol Oncol. 2016;9(1):51. 27363983 10.1186/s13045-016-0283-0 PMC4929758

[102] Lonial S , Weiss BM , Usmani SZ , et al. Daratumumab monotherapy in patients with treatment‐refractory multiple myeloma (SIRIUS): an open‐label, randomised, phase 2 trial. Lancet. 2016;387(10027):1551‐1560. 26778538 10.1016/S0140-6736(15)01120-4

[103] Usmani SZ , Weiss BM , Plesner T , et al. Clinical efficacy of daratumumab monotherapy in patients with heavily pretreated relapsed or refractory multiple myeloma. Blood. 2016;128(1):37‐44. 27216216 10.1182/blood-2016-03-705210 PMC4937359

[104] Palumbo A , Chanan‐Khan A , Weisel K , et al. Daratumumab, bortezomib, and dexamethasone for multiple myeloma. N Engl J Med. 2016;375(8):754‐766. 27557302 10.1056/NEJMoa1606038

[105] Mateos MV , Sonneveld P , Hungria V , et al. Daratumumab, bortezomib, and dexamethasone versus bortezomib and dexamethasone in patients with previously treated multiple myeloma: three‐year follow‐up of CASTOR. Clin Lymphoma Myeloma Leuk. 2020;20(8):509‐518. 32482541 10.1016/j.clml.2019.09.623

[106] Weisel K , Spencer A , Lentzsch S , et al. Daratumumab, bortezomib, and dexamethasone in relapsed or refractory multiple myeloma: subgroup analysis of CASTOR based on cytogenetic risk. J Hematol Oncol. 2020;13(1):115. 32819447 10.1186/s13045-020-00948-5 PMC7439722

[107] Bahlis NJ , Dimopoulos MA , White DJ , et al. Daratumumab plus lenalidomide and dexamethasone in relapsed/refractory multiple myeloma: extended follow‐up of POLLUX, a randomized, open‐label, phase 3 study. Leukemia. 2020;34(7):1875‐1884. 32001798 10.1038/s41375-020-0711-6 PMC7326710

[108] Mateos MV , Nahi H , Legiec W , et al. Subcutaneous versus intravenous daratumumab in patients with relapsed or refractory multiple myeloma (COLUMBA): a multicentre, open‐label, non‐inferiority, randomised, phase 3 trial. Lancet Haematol. 2020;7(5):e370‐e380. 32213342 10.1016/S2352-3026(20)30070-3

[109] Dhillon S . Isatuximab: first approval. Drugs. 2020;80(9):905‐912. 32347476 10.1007/s40265-020-01311-1

[110] Dimopoulos M , Bringhen S , Anttila P , et al. Isatuximab as monotherapy and combined with dexamethasone in patients with relapsed/refractory multiple myeloma. Blood. 2021;137(9):1154‐1165. 33080623 10.1182/blood.2020008209 PMC7933767

[111] Moreau P , Dimopoulos MA , Mikhael J , et al. Isatuximab, carfilzomib, and dexamethasone in relapsed multiple myeloma (IKEMA): a multicentre, open‐label, randomised phase 3 trial. Lancet. 2021;397(10292):2361‐2371. 34097854 10.1016/S0140-6736(21)00592-4

[112] Campbell KS , Cohen AD , Pazina T . Mechanisms of NK cell activation and clinical activity of the therapeutic SLAMF7 antibody, elotuzumab in multiple myeloma. Front Immunol. 2018;9:2551. 30455698 10.3389/fimmu.2018.02551 PMC6230619

[113] Passey C , Sheng J , Mora J , et al. The clinical pharmacology of elotuzumab. Clin Pharmacokinet. 2018;57(3):297‐313. 28779463 10.1007/s40262-017-0585-6

[114] Balasa B , Yun R , Belmar NA , et al. Elotuzumab enhances natural killer cell activation and myeloma cell killing through interleukin‐2 and TNF‐α pathways. Cancer Immunol Immunother. 2015;64(1):61‐73. 25287778 10.1007/s00262-014-1610-3 PMC4282702

[115] Dimopoulos MA , Dytfeld D , Grosicki S , et al. Elotuzumab plus pomalidomide and dexamethasone for multiple myeloma. N Engl J Med. 2018;379(19):1811‐1822. 30403938 10.1056/NEJMoa1805762

[116] Fulciniti M , Tassone P , Hideshima T , et al. Anti‐DKK1 mAb (BHQ880) as a potential therapeutic agent for multiple myeloma. Blood. 2009;114(2):371‐379. 19417213 10.1182/blood-2008-11-191577 PMC2714212

[117] Iyer SP , Beck JT , Stewart AK , et al. A Phase IB multicentre dose‐determination study of BHQ880 in combination with anti‐myeloma therapy and zoledronic acid in patients with relapsed or refractory multiple myeloma and prior skeletal‐related events. Br J Haematol. 2014;167(3):366‐375. 25139740 10.1111/bjh.13056

[118] Sadelain M , Brentjens R , Rivière I . The basic principles of chimeric antigen receptor design. Cancer Discov. 2013;3(4):388‐398. 23550147 10.1158/2159-8290.CD-12-0548 PMC3667586

[119] Savoldo B , Ramos CA , Liu E , et al. CD28 costimulation improves expansion and persistence of chimeric antigen receptor‐modified T cells in lymphoma patients. J Clin Invest. 2011;121(5):1822‐1826. 21540550 10.1172/JCI46110 PMC3083795

[120] Kochenderfer JN , Rosenberg SA . Treating B‐cell cancer with T cells expressing anti‐CD19 chimeric antigen receptors. Nat Rev Clin Oncol. 2013;10(5):267‐276. 23546520 10.1038/nrclinonc.2013.46 PMC6322669

[121] Gogishvili T , Danhof S , Prommersberger S , et al. SLAMF7‐CAR T cells eliminate myeloma and confer selective fratricide of SLAMF7(+) normal lymphocytes. Blood. 2017;130(26):2838‐2847. 29089311 10.1182/blood-2017-04-778423

[122] Garfall AL , Stadtmauer EA , Hwang WT , et al. Anti‐CD19 CAR T cells with high‐dose melphalan and autologous stem cell transplantation for refractory multiple myeloma. JCI Insight. 2018;3(8):e120505. 10.1172/jci.insight.120505 PMC5931130 29669947

[123] Cohen AD , Garfall AL , Stadtmauer EA , et al. B cell maturation antigen‐specific CAR T cells are clinically active in multiple myeloma. J Clin Invest. 2019;129(6):2210‐2221. 30896447 10.1172/JCI126397 PMC6546468

[124] Shah N , Chari A , Scott E , Mezzi K , Usmani SZ . B‐cell maturation antigen (BCMA) in multiple myeloma: rationale for targeting and current therapeutic approaches. Leukemia. 2020;34(4):985‐1005. 32055000 10.1038/s41375-020-0734-z PMC7214244

[125] Raje N , Berdeja J , Lin Y , et al. Anti‐BCMA CAR T‐cell therapy bb2121 in relapsed or refractory multiple myeloma. N Engl J Med. 2019;380(18):1726‐1737. 31042825 10.1056/NEJMoa1817226 PMC8202968

[126] Munshi NC , Anderson LD Jr. , Shah N , et al. Idecabtagene vicleucel in relapsed and refractory multiple myeloma. N Engl J Med. 2021;384(8):705‐716. 33626253 10.1056/NEJMoa2024850

[127] Berdeja JG , Madduri D , Usmani SZ , et al. Ciltacabtagene autoleucel, a B‐cell maturation antigen‐directed chimeric antigen receptor T‐cell therapy in patients with relapsed or refractory multiple myeloma (CARTITUDE‐1): a phase 1b/2 open‐label study. Lancet. 2021;398(10297):314‐324. 34175021 10.1016/S0140-6736(21)00933-8

[128] McClure JJ , Li X , Chou CJ . Advances and challenges of HDAC inhibitors in cancer therapeutics. Adv Cancer Res. 2018;138:183‐211. 29551127 10.1016/bs.acr.2018.02.006

[129] Ho M , Chen T , Liu J , et al. Targeting histone deacetylase 3 (HDAC3) in the bone marrow microenvironment inhibits multiple myeloma proliferation by modulating exosomes and IL‐6 trans‐signaling. Leukemia. 2020;34(1):196‐209. 31142847 10.1038/s41375-019-0493-x PMC6883144

[130] Bae J , Hideshima T , Tai YT , et al. Histone deacetylase (HDAC) inhibitor ACY241 enhances anti‐tumor activities of antigen‐specific central memory cytotoxic T lymphocytes against multiple myeloma and solid tumors. Leukemia. 2018;32(9):1932‐1947. 29487385 10.1038/s41375-018-0062-8 PMC6537609

[131] San‐Miguel JF , Hungria VT , Yoon SS , et al. Panobinostat plus bortezomib and dexamethasone versus placebo plus bortezomib and dexamethasone in patients with relapsed or relapsed and refractory multiple myeloma: a multicentre, randomised, double‐blind phase 3 trial. Lancet Oncol. 2014;15(11):1195‐1206. 25242045 10.1016/S1470-2045(14)70440-1

[132] Laubach JP , Schjesvold F , Mariz M , et al. Efficacy and safety of oral panobinostat plus subcutaneous bortezomib and oral dexamethasone in patients with relapsed or relapsed and refractory multiple myeloma (PANORAMA 3): an open‐label, randomised, phase 2 study. Lancet Oncol. 2021;22(1):142‐154. 33301738 10.1016/S1470-2045(20)30680-X

[133] Berdeja JG , Gregory TK , Faber EA , et al. A phase I/II study of the combination of panobinostat and carfilzomib in patients with relapsed or relapsed/refractory multiple myeloma: final analysis of second dose‐expansion cohort. Am J Hematol. 2021;96(4):428‐435. 33421178 10.1002/ajh.26088 PMC7986798

[134] Vogl DT , Raje N , Jagannath S , et al. Ricolinostat, the first selective histone deacetylase 6 inhibitor, in combination with bortezomib and dexamethasone for relapsed or refractory multiple myeloma. Clin Cancer Res. 2017;23(13):3307‐3315. 28053023 10.1158/1078-0432.CCR-16-2526 PMC5496796

[135] Delbridge AR , Strasser A . The BCL‐2 protein family, BH3‐mimetics and cancer therapy. Cell Death Differ. 2015;22(7):1071‐1080. 25952548 10.1038/cdd.2015.50 PMC4572872

[136] Touzeau C , Maciag P , Amiot M , Moreau P . Targeting Bcl‐2 for the treatment of multiple myeloma. Leukemia. 2018;32(9):1899‐1907. 30076373 10.1038/s41375-018-0223-9

[137] Kumar SK , Harrison SJ , Cavo M , et al. Venetoclax or placebo in combination with bortezomib and dexamethasone in patients with relapsed or refractory multiple myeloma (BELLINI): a randomised, double‐blind, multicentre, phase 3 trial. Lancet Oncol. 2020;21(12):1630‐1642. 33129376 10.1016/S1470-2045(20)30525-8

[138] Kaufman JL , Gasparetto C , Schjesvold FH , et al. Targeting BCL‐2 with venetoclax and dexamethasone in patients with relapsed/refractory t(11;14) multiple myeloma. Am J Hematol. 2021;96(4):418‐427. 33368455 10.1002/ajh.26083 PMC7986778

[139] Azmi AS , Uddin MH , Mohammad RM . The nuclear export protein XPO1: from biology to targeted therapy. Nat Rev Clin Oncol. 2021;18(3):152‐169. 33173198 10.1038/s41571-020-00442-4

[140] Richard S , Richter J , Jagannath S . Selinexor: a first‐in‐class SINE compound for treatment of relapsed refractory multiple myeloma. Future Oncol. 2020;16(19):1331‐1350. 32511022 10.2217/fon-2020-0054

[141] Chari A , Vogl DT , Gavriatopoulou M , et al. Oral selinexor‐dexamethasone for triple‐class refractory multiple myeloma. N Engl J Med. 2019;381(8):727‐738. 31433920 10.1056/NEJMoa1903455

[142] Grosicki S , Simonova M , Spicka I , et al. Once‐per‐week selinexor, bortezomib, and dexamethasone versus twice‐per‐week bortezomib and dexamethasone in patients with multiple myeloma (BOSTON): a randomised, open‐label, phase 3 trial. Lancet. 2020;396(10262):1563‐1573. 33189178 10.1016/S0140-6736(20)32292-3

[143] Jakubowiak AJ , Jasielec JK , Rosenbaum CA , et al. Phase 1 study of selinexor plus carfilzomib and dexamethasone for the treatment of relapsed/refractory multiple myeloma. Br J Haematol. 2019;186(4):549‐560. 31124580 10.1111/bjh.15969 PMC6772147

[144] Yu WD , Sun G , Li J , Xu J , Wang X . Mechanisms and therapeutic potentials of cancer immunotherapy in combination with radiotherapy and/or chemotherapy. Cancer Lett. 2019;452:66‐70. 30902563 10.1016/j.canlet.2019.02.048

[145] Topp MS , Duell J , Zugmaier G , et al. Anti‐B‐cell maturation antigen BiTE molecule AMG 420 induces responses in multiple myeloma. J Clin Oncol. 2020;38(8):775‐783. 31895611 10.1200/JCO.19.02657

[146] Usmani SZ , Garfall AL , van de Donk N , et al. Teclistamab, a B‐cell maturation antigen × CD3 bispecific antibody, in patients with relapsed or refractory multiple myeloma (MajesTEC‐1): a multicentre, open‐label, single‐arm, phase 1 study. Lancet. 2021;398(10301):665‐674. 34388396 10.1016/S0140-6736(21)01338-6

[147] Nishida H , Yamada T . Monoclonal antibody therapies in multiple myeloma: a challenge to develop novel targets. J Oncol. 2019;2019:6084012. 31781214 10.1155/2019/6084012 PMC6875016

[148] Lonial S , Lee HC , Badros A , et al. Belantamab mafodotin for relapsed or refractory multiple myeloma (DREAMM‐2): a two‐arm, randomised, open‐label, phase 2 study. Lancet Oncol. 2020;21(2):207‐221. 31859245 10.1016/S1470-2045(19)30788-0

[149] Ghilardi G , Pabst T , Jeker B , et al. Melphalan dose in myeloma patients ≥65 years of age undergoing high‐dose therapy and autologous stem cell transplantation: a multicentric observational registry study. Bone Marrow Transplant. 2019;54(7):1029‐1037. 30390061 10.1038/s41409-018-0379-y

[150] Brioli A , Vom Hofe F , Rucci P , et al. Melphalan 200 mg/m(2) does not increase toxicity and improves survival in comparison to reduced doses of melphalan in multiple myeloma patients. Bone Marrow Transplant. 2021;56(5):1209‐1212. 33299059 10.1038/s41409-020-01170-0

[151] Saini N , Bashir Q , Milton DR , et al. Busulfan and melphalan conditioning is superior to melphalan alone in autologous stem cell transplantation for high‐risk MM. Blood Adv. 2020;4(19):4834‐4837. 33027527 10.1182/bloodadvances.2020002590 PMC7556152

[152] Attal M , Lauwers‐Cances V , Hulin C , et al. Lenalidomide, bortezomib, and dexamethasone with transplantation for myeloma. N Engl J Med. 2017;376(14):1311‐1320. 28379796 10.1056/NEJMoa1611750 PMC6201242

[153] Dhakal B , Szabo A , Chhabra S , et al. Autologous transplantation for newly diagnosed multiple myeloma in the era of novel agent induction: a systematic review and meta‐analysis. JAMA Oncol. 2018;4(3):343‐350. 29302684 10.1001/jamaoncol.2017.4600 PMC5885822

[154] Chim CS , Kumar SK , Orlowski RZ , et al. Management of relapsed and refractory multiple myeloma: novel agents, antibodies, immunotherapies and beyond. Leukemia. 2018;32(2):252‐262. 29257139 10.1038/leu.2017.329 PMC5808071

[155] Liao J , Jia Y , Wu Y , et al. Physical‐, chemical‐, and biological‐responsive nanomedicine for cancer therapy. Wiley Interdiscip Rev Nanomed Nanobiotechnol. 2020;12(1):e1581. 31429208 10.1002/wnan.1581

[156] Kim BY , Rutka JT , Chan WC . Nanomedicine. N. Engl. J. Med. 2010;363(25):2434‐2443. 21158659 10.1056/NEJMra0912273

[157] Freitas RA Jr . What is nanomedicine? Nanomedicine. 2005;1(1):2‐9. 17292052 10.1016/j.nano.2004.11.003

[158] Bayda S , Adeel M , Tuccinardi T , Cordani M , Rizzolio F . The history of nanoscience and nanotechnology: from chemical‐physical applications to nanomedicine. Molecules. 2019;25(1):112. 10.3390/molecules25010112 PMC6982820 31892180

[159] Han RX , Peng JR , Xiao Y , Hao Y , Jia YP , Qian ZY . Ag_2_S nanoparticles as an emerging single‐component theranostic agent. Chin Chem Lett. 2020;31(7):1717‐1728.

[160] Yu X , Deng XH , Qian WR , Luo XY , He QH , He J . Synergistic combination of polymer‐coated copper and selenium nanoparticles and X‐ray induced radiotherapy for improved nursing care of human prostate cancer cells. Mater Express. 2021;11(3):287‐295.

[161] Zheleznyak A , Shokeen M , Achilefu S . Nanotherapeutics for multiple myeloma. Wiley Interdiscip Rev Nanomed Nanobiotechnol. 2018;10(6):e1526. 29701006 10.1002/wnan.1526 PMC6185771

[162] Wang M , Qu Y , Hu D , Niu T , Qian Z . Nanomedicine applications in treatment of primary central nervous system lymphoma: current state of the art. J Biomed Nanotechnol. 2021;17(8):1459‐1485. 34544527 10.1166/jbn.2021.3133

[163] Danhier F . To exploit the tumor microenvironment: since the EPR effect fails in the clinic, what is the future of nanomedicine? J Control Release. 2016;244(Pt A):108‐121. 27871992 10.1016/j.jconrel.2016.11.015

[164] Prabhakar U , Maeda H , Jain RK , et al. Challenges and key considerations of the enhanced permeability and retention effect for nanomedicine drug delivery in oncology. Cancer Res. 2013;73(8):2412‐2417. 23423979 10.1158/0008-5472.CAN-12-4561 PMC3916009

[165] Detappe A , Bustoros M , Mouhieddine TH , Ghoroghchian PP . Advancements in nanomedicine for multiple myeloma. Trends Mol Med. 2018;24(6):560‐574. 29773319 10.1016/j.molmed.2018.04.005

[166] Zhong W , Zhang X , Zhao M , Wu J , Lin D . Advancements in nanotechnology for the diagnosis and treatment of multiple myeloma. Biomater Sci. 2020;8(17):4692‐4711. 32779645 10.1039/d0bm00772b

[167] Arranja AG , Pathak V , Lammers T , Shi Y . Tumor‐targeted nanomedicines for cancer theranostics. Pharmacol Res. 2017;115:87‐95. 27865762 10.1016/j.phrs.2016.11.014 PMC5412956

[168] Wang D , Liu W , Wang L , et al. Suppression of cancer proliferation and metastasis by a versatile nanomedicine integrating photodynamic therapy, photothermal therapy, and enzyme inhibition. Acta Biomater. 2020;113:541‐553. 32562802 10.1016/j.actbio.2020.06.021

[169] Sengupta S . Cancer nanomedicine: lessons for immuno‐oncology. Trends Cancer. 2017;3(8):551‐560. 28780932 10.1016/j.trecan.2017.06.006

[170] Caracciolo G , Palchetti S , Digiacomo L , et al. Human biomolecular corona of liposomal doxorubicin: the overlooked factor in anticancer drug delivery. ACS Appl Mater Interfaces. 2018;10(27):22951‐22962. 29905462 10.1021/acsami.8b04962

[171] Gabizon AA , Patil Y , La‐Beck NM . New insights and evolving role of pegylated liposomal doxorubicin in cancer therapy. Drug Resist Updat. 2016;29:90‐106. 27912846 10.1016/j.drup.2016.10.003

[172] Deshantri AK , Fens MH , Ruiter RWJ , et al. Liposomal dexamethasone inhibits tumor growth in an advanced human‐mouse hybrid model of multiple myeloma. J Control Release. 2019;296:232‐240. 30682443 10.1016/j.jconrel.2019.01.028

[173] Celia C , Malara N , Terracciano R , et al. Liposomal delivery improves the growth‐inhibitory and apoptotic activity of low doses of gemcitabine in multiple myeloma cancer cells. Nanomedicine. 2008;4(2):155‐166. 18430611 10.1016/j.nano.2008.02.003

[174] Ashley JD , Stefanick JF , Schroeder VA , Suckow MA , Kiziltepe T , Bilgicer B . Liposomal bortezomib nanoparticles via boronic ester prodrug formulation for improved therapeutic efficacy in vivo. J Med Chem. 2014;57(12):5282‐5292. 24897555 10.1021/jm500352v

[175] Ashley JD , Stefanick JF , Schroeder VA , et al. Liposomal carfilzomib nanoparticles effectively target multiple myeloma cells and demonstrate enhanced efficacy in vivo. J Control Release. 2014;196:113‐121. 25312543 10.1016/j.jconrel.2014.10.005

[176] Landgren O , Iskander K . Modern multiple myeloma therapy: deep, sustained treatment response and good clinical outcomes. J Intern Med. 2017;281(4):365‐382. 28205262 10.1111/joim.12590

[177] Ashley JD , Quinlan CJ , Schroeder VA , et al. Dual carfilzomib and doxorubicin‐loaded liposomal nanoparticles for synergistic efficacy in multiple myeloma. Mol Cancer Ther. 2016;15(7):1452‐1459. 27196779 10.1158/1535-7163.MCT-15-0867

[178] Omstead DT , Mejia F , Sjoerdsma J , et al. In vivo evaluation of CD38 and CD138 as targets for nanoparticle‐based drug delivery in multiple myeloma. J Hematol Oncol. 2020;13(1):145. 33138841 10.1186/s13045-020-00965-4 PMC7607744

[179] Chang Q , Geng R , Wang S , Qu D , Kong X . DOPA‐based paclitaxel‐loaded liposomes with modifications of transferrin and alendronate for bone and myeloma targeting. Drug Deliv. 2016;23(9):3629‐3638. 27749106 10.1080/10717544.2016.1214989

[180] Stefanick JF , Omstead DT , Kiziltepe T , Bilgicer B . Dual‐receptor targeted strategy in nanoparticle design achieves tumor cell selectivity through cooperativity. Nanoscale. 2019;11(10):4414‐4427. 30801591 10.1039/c8nr09431d

[181] Federico C , Alhallak K , Sun J , et al. Tumor microenvironment‐targeted nanoparticles loaded with bortezomib and ROCK inhibitor improve efficacy in multiple myeloma. Nat Commun. 2020;11(1):6037. 33247158 10.1038/s41467-020-19932-1 PMC7699624

[182] Azab AK , Azab F , Blotta S , et al. RhoA and Rac1 GTPases play major and differential roles in stromal cell‐derived factor‐1‐induced cell adhesion and chemotaxis in multiple myeloma. Blood. 2009;114(3):619‐629. 19443661 10.1182/blood-2009-01-199281 PMC2713475

[183] Alhallak K , Sun J , Wasden K , et al. Nanoparticle T‐cell engagers as a modular platform for cancer immunotherapy. Leukemia. 2021;35(8):2346‐2357. 33479469 10.1038/s41375-021-01127-2 PMC8292428

[184] Ao L , Reichel D , Hu D , et al. Polymer micelle formulations of proteasome inhibitor carfilzomib for improved metabolic stability and anticancer efficacy in human multiple myeloma and lung cancer cell lines. J Pharmacol Exp Ther. 2015;355(2):168‐173. 26311812 10.1124/jpet.115.226993 PMC4613964

[185] Zhang C , Wang X , Cheng R , Zhong Z . A6 peptide‐tagged core‐disulfide‐cross‐linked micelles for targeted delivery of proteasome inhibitor carfilzomib to multiple myeloma in vivo. Biomacromolecules. 2020;21(6):2049‐2059. 32338875 10.1021/acs.biomac.9b01790

[186] Fontana F , Scott MJ , Allen JS , et al. VLA4‐targeted nanoparticles hijack cell adhesion‐mediated drug resistance to target refractory myeloma cells and prolong survival. Clin Cancer Res. 2021;27(7):1974‐1986. 33355244 10.1158/1078-0432.CCR-20-2839 PMC8026499

[187] Seelan RS , Mukhopadhyay P , Pisano MM , Greene RM . Effects of 5‐Aza‐2'‐deoxycytidine (decitabine) on gene expression. Drug Metab Rev. 2018;50(2):193‐207. 29455551 10.1080/03602532.2018.1437446

[188] Che F , Chen J , Dai J , Liu X . Inhibition of multiple myeloma using 5‐aza‐2'‐deoxycytidine and bortezomib‐loaded self‐assembling nanoparticles. Cancer Manag Res. 2020;12:6969‐6976. 32848460 10.2147/CMAR.S255682 PMC7425094

[189] Gu Z , Wang X , Cheng R , Cheng L , Zhong Z . Hyaluronic acid shell and disulfide‐crosslinked core micelles for in vivo targeted delivery of bortezomib for the treatment of multiple myeloma. Acta Biomater. 2018;80:288‐295. 30240956 10.1016/j.actbio.2018.09.022

[190] Zhong Y , Meng F , Deng C , Mao X , Zhong Z . Targeted inhibition of human hematological cancers in vivo by doxorubicin encapsulated in smart lipoic acid‐crosslinked hyaluronic acid nanoparticles. Drug Deliv. 2017;24(1):1482‐1490. 28958164 10.1080/10717544.2017.1384864 PMC8240992

[191] Tai YT , Anderson KC . B cell maturation antigen (BCMA)‐based immunotherapy for multiple myeloma. Expert Opin Biol Ther. 2019;19(11):1143‐1156. 31277554 10.1080/14712598.2019.1641196 PMC6785394

[192] Bae J , Parayath N , Ma W , Amiji M , Munshi N , Anderson KC . BCMA peptide‐engineered nanoparticles enhance induction and function of antigen‐specific CD8(+) cytotoxic T lymphocytes against multiple myeloma: clinical applications. Leukemia. 2020;34(1):210‐223. 31427721 10.1038/s41375-019-0540-7 PMC7297141

[193] Guo S , Xiao P , Li B , et al. Co‐immunizing with PD‐L1 induces CD8(+) DCs‐mediated anti‐tumor immunity in multiple myeloma. Int Immunopharmacol. 2020;84:106516. 32334387 10.1016/j.intimp.2020.106516

[194] Gu W , An J , Meng H , et al. CD44‐specific A6 short peptide boosts targetability and anticancer efficacy of polymersomal epirubicin to orthotopic human multiple myeloma. Adv Mater. 2019;31(46):e1904742. 31560141 10.1002/adma.201904742

[195] Zhong Y , Meng F , Zhang W , Li B , van Hest JCM , Zhong Z . CD44‐targeted vesicles encapsulating granzyme B as artificial killer cells for potent inhibition of human multiple myeloma in mice. J Control Release. 2020;320:421‐430. 32027936 10.1016/j.jconrel.2020.02.004

[196] Yu N , Zhang Y , Li J , et al. Daratumumab immunopolymersome‐enabled safe and CD38‐targeted chemotherapy and depletion of multiple myeloma. Adv Mater. 2021;33(39):e2007787. 34369013 10.1002/adma.202007787

[197] de la Puente P , Luderer MJ , Federico C , et al. Enhancing proteasome‐inhibitory activity and specificity of bortezomib by CD38 targeted nanoparticles in multiple myeloma. J Control Release. 2018;270:158‐176. 29196043 10.1016/j.jconrel.2017.11.045 PMC6056271

[198] Huang YH , Vakili MR , Molavi O , et al. Decoration of anti‐CD38 on nanoparticles carrying a STAT3 inhibitor can improve the therapeutic efficacy against myeloma. Cancers (Basel). 2019;11(2):248. 10.3390/cancers11020248 PMC6407065 30791634

[199] Mhaskar R , Kumar A , Miladinovic B , Djulbegovic B . Bisphosphonates in multiple myeloma: an updated network meta‐analysis. Cochrane Database Syst Rev. 2017;12(12):Cd003188. 29253322 10.1002/14651858.CD003188.pub4 PMC6486151

[200] Swami A , Reagan MR , Basto P , et al. Engineered nanomedicine for myeloma and bone microenvironment targeting. Proc Natl Acad Sci U S A. 2014;111(28):10287‐10292. 24982170 10.1073/pnas.1401337111 PMC4104924

[201] Zhang W , Qiao L , Wang X , Senthilkumar R , Wang F , Chen B . Inducing cell cycle arrest and apoptosis by dimercaptosuccinic acid modified Fe3O4 magnetic nanoparticles combined with nontoxic concentration of bortezomib and gambogic acid in RPMI‐8226 cells. Int J Nanomedicine. 2015;10:3275‐3289. 25995634 10.2147/IJN.S80795 PMC4425315

[202] Howat S , Park B , Oh IS , Jin YW , Lee EK , Loake GJ . Paclitaxel: biosynthesis, production and future prospects. N Biotechnol. 2014;31(3):242‐245. 24614567 10.1016/j.nbt.2014.02.010

[203] Yang C , Wang J , Chen D , et al. Paclitaxel‐Fe3O4 nanoparticles inhibit growth of CD138(‐) CD34(‐) tumor stem‐like cells in multiple myeloma‐bearing mice. Int J Nanomedicine. 2013;8:1439‐1449. 23610522 10.2147/IJN.S38447 PMC3629869

[204] Gobbo OL , Sjaastad K , Radomski MW , Volkov Y , Prina‐Mello A . Magnetic nanoparticles in cancer theranostics. Theranostics. 2015;5(11):1249‐1263. 26379790 10.7150/thno.11544 PMC4568452

[205] Hayashi K , Nakamura M , Sakamoto W , et al. Superparamagnetic nanoparticle clusters for cancer theranostics combining magnetic resonance imaging and hyperthermia treatment. Theranostics. 2013;3(6):366‐376. 23781284 10.7150/thno.5860 PMC3677408

[206] Wright GEB , Amstutz U , Drögemöller BI , et al. Pharmacogenomics of vincristine‐induced peripheral neuropathy implicates pharmacokinetic and inherited neuropathy genes. Clin Pharmacol Ther. 2019;105(2):402‐410. 29999516 10.1002/cpt.1179 PMC6519044

[207] Patra HK , Dasgupta AK , Sarkar S , Biswas I , Chattopadhyay A . Dual role of nanoparticles as drug carrier and drug. Cancer Nanotechnol. 2011;2(1‐6):37‐47. 26069483 10.1007/s12645-010-0011-3 PMC4451630

[208] Abou DS , Thorek DL , Ramos NN , et al. (89)Zr‐labeled paramagnetic octreotide‐liposomes for PET‐MR imaging of cancer. Pharm Res. 2013;30(3):878‐888. 23224977 10.1007/s11095-012-0929-8 PMC3578092

[209] Tang R , Zheleznyak A , Mixdorf M , et al. Osteotropic radiolabeled nanophotosensitizer for imaging and treating multiple myeloma. ACS Nano. 2020;14(4):4255‐4264. 32223222 10.1021/acsnano.9b09618 PMC7295119

[210] Morelli C , Maris P , Sisci D , et al. PEG‐templated mesoporous silica nanoparticles exclusively target cancer cells. Nanoscale. 2011;3(8):3198‐3207. 21725561 10.1039/c1nr10253b

[211] Nigro A , Frattaruolo L , Fava M , et al. Bortezomib‐loaded mesoporous silica nanoparticles selectively alter metabolism and induce death in multiple myeloma cells. Cancers (Basel). 2020;12(9):2709. 10.3390/cancers12092709 PMC7565423 32967380

[212] Dias AP , da Silva Santos S , da Silva JV , et al. Dendrimers in the context of nanomedicine. Int J Pharm. 2020;573:118814. 31759101 10.1016/j.ijpharm.2019.118814

[213] Chaudhary S , Gothwal A , Khan I , Srivastava S , Malik R , Gupta U . Polypropyleneimine and polyamidoamine dendrimer mediated enhanced solubilization of bortezomib: comparison and evaluation of mechanistic aspects by thermodynamics and molecular simulations. Mater Sci Eng C Mater Biol Appl. 2017;72:611‐619. 28024628 10.1016/j.msec.2016.11.122

[214] Hofland T , Eldering E , Kater AP , Tonino SH . Engaging cytotoxic T and NK cells for immunotherapy in chronic lymphocytic leukemia. Int J Mol Sci. 2019;20(17):4315. 10.3390/ijms20174315 PMC6747204 31484424

[215] Poupot M , Turrin CO , Caminade AM , et al. Poly(phosphorhydrazone) dendrimers: yin and yang of monocyte activation for human NK cell amplification applied to immunotherapy against multiple myeloma. Nanomedicine. 2016;12(8):2321‐2330. 27498187 10.1016/j.nano.2016.07.009

[216] Ma N , Yan Z . Research progress of thermosensitive hydrogel in tumor therapeutic. Nanoscale Res Lett. 2021;16(1):42. 33665739 10.1186/s11671-021-03502-5 PMC7933296

[217] Jiang Y , Krishnan N , Heo J , Fang RH , Zhang L . Nanoparticle‐hydrogel superstructures for biomedical applications. J Control Release. 2020;324:505‐521. 32464152 10.1016/j.jconrel.2020.05.041 PMC7429280

[218] Lee ALZ , Voo ZX , Chin W , et al. Injectable coacervate hydrogel for delivery of anticancer drug‐loaded nanoparticles in vivo. ACS Appl Mater Interfaces. 2018;10(16):13274‐13282. 29595244 10.1021/acsami.7b14319

[219] Chen HY , Deng J , Wang Y , Wu CQ , Li X , Dai HW . Hybrid cell membrane‐coated nanoparticles: a multifunctional biomimetic platform for cancer diagnosis and therapy. Acta Biomater. 2020;112:1‐13. 32470527 10.1016/j.actbio.2020.05.028

[220] Oroojalian F , Beygi M , Baradaran B , Mokhtarzadeh A , Shahbazi MA . Immune cell membrane‐coated biomimetic nanoparticles for targeted cancer therapy. Small. 2021;17(12):e2006484. 33577127 10.1002/smll.202006484

[221] Hu Q , Qian C , Sun W , et al. Engineered nanoplatelets for enhanced treatment of multiple myeloma and thrombus. Adv Mater. 2016;28(43):9573‐9580. 27626769 10.1002/adma.201603463 PMC5283718

[222] Qu Y , Chu B , Wei X , et al. Cancer‐cell‐biomimetic nanoparticles for targeted therapy of multiple myeloma based on bone marrow homing. Adv Mater. 2021:e2107883. 34877715 10.1002/adma.202107883

[223] Spring BQ , Bryan Sears R , Zheng LZ , et al. A photoactivable multi‐inhibitor nanoliposome for tumour control and simultaneous inhibition of treatment escape pathways. Nat Nanotechnol. 2016;11(4):378‐387. 26780659 10.1038/nnano.2015.311 PMC4821671

[224] Shen LJ , Zhou TJ , Fan YT , et al. Recent progress in tumor photodynamic immunotherapy. Chin Chem Lett. 2020;31(7):1709‐1716.

[225] Kotagiri N , Cooper ML , Rettig M , et al. Radionuclides transform chemotherapeutics into phototherapeutics for precise treatment of disseminated cancer. Nat Commun. 2018;9(1):275. 29348537 10.1038/s41467-017-02758-9 PMC5773683

[226] Shi F , Li M , Wu S , et al. Enhancing the anti‐multiple myeloma efficiency in a cancer stem cell xenograft model by conjugating the ABCG2 antibody with microbubbles for a targeted delivery of ultrasound mediated epirubicin. Biochem Pharmacol. 2017;132:18‐28. 28232025 10.1016/j.bcp.2017.02.014

[227] Harris L , Batist G , Belt R , et al. Liposome‐encapsulated doxorubicin compared with conventional doxorubicin in a randomized multicenter trial as first‐line therapy of metastatic breast carcinoma. Cancer. 2002;94(1):25‐36. 11815957 10.1002/cncr.10201

[228] Orlowski RZ , Nagler A , Sonneveld P , et al. Randomized phase III study of pegylated liposomal doxorubicin plus bortezomib compared with bortezomib alone in relapsed or refractory multiple myeloma: combination therapy improves time to progression. J Clin Oncol. 2007;25(25):3892‐3901. 17679727 10.1200/JCO.2006.10.5460

[229] Orlowski RZ , Nagler A , Sonneveld P , et al. Final overall survival results of a randomized trial comparing bortezomib plus pegylated liposomal doxorubicin with bortezomib alone in patients with relapsed or refractory multiple myeloma. Cancer. 2016;122(13):2050‐2056. 27191689 10.1002/cncr.30026 PMC5701574

[230] Wang Q , Tardi P , Sadowski N , Xie S , Heller D , Mayer L . Pharmacokinetics, drug metabolism, and tissue distribution of CPX‐351 in animals. Nanomedicine. 2020;30:102275. 32750494 10.1016/j.nano.2020.102275

[231] Cortes JE , Lin TL , Uy GL , Ryan RJ , Faderl S , Lancet JE . Quality‐adjusted time without symptoms of disease or toxicity (Q‐TWiST) analysis of CPX‐351 versus 7 + 3 in older adults with newly diagnosed high‐risk/secondary AML. J Hematol Oncol. 2021;14(1):110. 34256819 10.1186/s13045-021-01119-w PMC8276472

[232] Stefanick JF , Ashley JD , Kiziltepe T , Bilgicer B . A systematic analysis of peptide linker length and liposomal polyethylene glycol coating on cellular uptake of peptide‐targeted liposomes. ACS Nano. 2013;7(4):2935‐2947. 23421406 10.1021/nn305663e

[233] Bar‐Zeev M , Livney YD , Assaraf YG . Targeted nanomedicine for cancer therapeutics: towards precision medicine overcoming drug resistance. Drug Resist Updat. 2017;31:15‐30. 28867241 10.1016/j.drup.2017.05.002

[234] Huang SY , Tien HF , Su FH , Hsu SM . Nonirradiated NOD/SCID‐human chimeric animal model for primary human multiple myeloma: a potential in vivo culture system. Am J Pathol. 2004;164(2):747‐756. 14742278 10.1016/S0002-9440(10)63162-8 PMC1602249

[235] Alici E , Konstantinidis KV , Aints A , Dilber MS , Abedi‐Valugerdi M . Visualization of 5T33 myeloma cells in the C57BL/KaLwRij mouse: establishment of a new syngeneic murine model of multiple myeloma. Exp Hematol. 2004;32(11):1064‐1072. 15539084 10.1016/j.exphem.2004.07.019

